# DIA-Estimator and Multidimensional Model Misspecifications: GNSS-based Positioning Safety Analysis for UAVs

**DOI:** 10.1007/s10291-026-02029-5

**Published:** 2026-03-09

**Authors:** Sebastian Ciuban, Peter J. G. Teunissen, Christian C. J. M. Tiberius

**Affiliations:** 1 Department of Geoscience and Remote Sensing, Delft University of Technology, Delft, The Netherlands; 2 Department of Infrastructure Engineering, University of Melbourne, Melbourne, Australia; 3 GNSS Research Centre, Curtin University, Perth, Australia

**Keywords:** DIA-Estimator, Positioning safety, Multidimensional model misspecifications, Probability of positioning failure

## Abstract

The Detection, Identification, and Adaptation (DIA)-estimator integrates parameter estimation and hypothesis testing for model misspecifications. This contribution presents a positioning safety analysis approach grounded in the DIA-estimator framework, with a particular emphasis on multidimensional model misspecifications, such as simultaneous outliers in the observations. While recent work has focused on the performance of the detection and identification of multidimensional model misspecifications, we turn our attention to how they affect the probability density function (PDF) of the DIA-estimator and, consequently, the probability of positioning failure–an indicator relevant for safety-of-life applications (e.g., automotive, aviation, rail, maritime). This work formulates and quantifies the probability of positioning failure and its conditional components. A representative simulation-based study is presented for a UAV equipped with a GPS receiver configured to achieve performance comparable to Technical Standard Order (TSO)-certified receivers. The analysis is carried out for two scenarios: a fixed GPS satellite geometry at a single time snapshot, and for a varying GPS satellite geometry over a 24-hour period over an authorized UAV airspace region in the Netherlands using real satellite ephemeris data. Together, these scenarios provide insights into the structure of the DIA-estimator’s PDF, such as multimodality and orientation with respect to the chosen positioning safety region, and support comprehensive evaluation of positioning safety. Although the current focus is on GPS-based positioning, the presented approach is general and can be extended to include multisensor configurations, additional GNSS constellations, and applied to other safety-critical applications, which are subjects of future work.

## Introduction

The Detection, Identification, and Adaptation (DIA)-estimator $\overline{\underline{\text {x}}} \in \mathbb {R}^n$, introduced by Teunissen, is part of a unifying framework that captures the interplay between parameter estimation and hypothesis testing in the context of the DIA method (Teunissen 2018). Parameter estimation is performed to obtain estimates of the parameters of interest, while hypothesis testing is used to validate these results and adapt the underlying models in the case of misspecifications. The DIA method has found applications across a wide range of domains, such as in GNSS-based positioning (Bakker and Tiberius 2017; Zaminpardaz et al. 2019; Ciuban et al. 2024) quality control in geodetic networks (DGCC 1984), deformation monitoring (Zaminpardaz et al. 2020), quality control in navigation system (Teunissen 1990), and, more recently, in positioning safety analyses for automated and autonomous driving (Ciuban et al. 2025a), among others.

Accounting for the occurrence of multidimensional misspecifications (e.g., multiple outliers) when deciding on the alternative models (hypotheses) for the DIA method is of particular interest, as it more accurately reflects real-world conditions. Recent studies have investigated the performance of the testing procedure with respect to its ability to detect and identify multiple outliers (Zaminpardaz and Teunissen 2023; Yu et al. 2023, 2024). However, these studies have primarily focused on the detection and identification aspects, without considering how such multidimensional misspecifications affect the probability density function (PDF) $f_{\overline{\underline{\text {x}}}}(x)$ of the DIA-estimator. This consideration is particularly important for positioning safety analyses in safety-of-life applications (e.g., automotive, aviation, rail, maritime), where the probability of positioning failure is of interest (Ciuban et al. 2024, 2025, 2025)

1 $$\mathbb {P}_{\mathcal {F}} = \textsf{P}\left( \overline{\underline{\mathrm{x}}} \in \mathcal {B}^c \right) =\int _{\mathcal {B}^c} f_{\overline{\underline{\text {x}}}}(x) d \textit{x}, $$

with $\mathcal {F} = \{\overline{\underline{\text {x}}} \in \mathcal {B}^c\}$ being the event of positioning failure (Special Committee 159, R.T.C.A. 2020) and $\mathcal {B}^c \subset \mathbb {R}^n$ is the complement of an application specific safety-region $\mathcal {B} \subset \mathbb {R}^n$. In this contribution, we present an approach for performing positioning safety analyses aimed at quantifying (1) and its conditional components, while accounting for multidimensional model misspecifications caused by outliers in the observations.

With respect to our previous research (Ciuban et al. 2024, 2025a, 2025b), the main contributions are as follows: (i) analysis of the impact of multidimensional outliers on the shape of the conditional components of the DIA-estimator’s $f_{\overline{\underline{\text {x}}}}(x)$; (ii) consideration of time-varying positioning functional and stochastic models (e.g., due to the evolution of GPS satellite geometry over 24 h) into the computation of the probability of positioning failure; (iii) application of the DIA estimator’s framework to Unmanned Aerial Vehicle (UAV) cases, demonstrating its relevance in this domain.

To illustrate the approach for positioning safety analysis, we present a representative case study for an Unmanned Aerial Vehicle (UAV) assumed to be equipped with a GPS receiver configured to achieve positioning performance comparable to that of receivers certified under the Technical Standard Order (TSO) for GPS-based UAV operations (FAA 2017; Cozzens 2021). The analysis is carried out for two scenarios. The first scenario considers a GPS satellite geometry as observed by the UAV’s GPS receiver at a snapshot of time, enabling detailed analysis of the conditional components of the DIA-estimator’s PDF relative to the shape of the safety region $\mathcal {B} \subset \mathbb {R}^n$, as well as the computed components of the probability of positioning failure. In the second scenario we consider a 24-hours (on May 24, 2024) evolution of GPS satellites moving over an airspace region in The Netherlands for which authorization can be obtained for UAV operations (EASA online). Together, these scenarios provide insights into the behavior of the DIA-estimator’s PDF and, consequently, into the components of the probability of positioning failure–supporting a comprehensive evaluation of positioning safety. The presented positioning safety analysis is consistent with scenario-based safety assessment frameworks, which are widely used in domains such as automated and autonomous vehicles (Riedmaier 2020; United Nations Economic Commission for Europe 2023; Gelder 2024) and UAVs (Khatiri 2023), among others.

This contribution is organized as follows: In Section 2 we briefly review the main principles of the DIA-estimator $\overline{\underline{\text {x}}} \in \mathbb {R}^n$ and of its PDF $f_{\overline{\underline{\text {x}}}}(x)$. In Section 3 we present the formulation of the probability of positioning failure and its components. Section 4 presents the positioning-safety analysis for a UAV at a single snapshot of time and over a period of 24 h. Section 6 contains the summary and the concluding remarks of this contribution. Throughout the paper we make use of the following notation: an underscore denotes a random quantity (e.g., the random vector $\underline{\text {y}} \in \mathbb {R}^m$), $f_{\underline{\text {y}}}(\textit{y})$ is the PDF of $\underline{\text {y}}\in \mathbb {R}^m$, and $\textsf{E}_{f_{\underline{\mathrm{y}}}}\left( \underline{\mathrm{y}} \right) = \int _{\mathbb {R}^m} \mathrm{y} f_{\underline{\mathrm{y}}}(\textit{y}) d\textit{y}$ is the expected value of $\underline{\text {y}}\in \mathbb {R}^m$. The joint PDF of two random vectors $\underline{\text {x}} \in \mathbb {R}^n$ and $\underline{\text {y}} \in \mathbb {R}^m$ is denoted $f_{\underline{\text {x}},\underline{\text {y}}}(x,y)$. A projection matrix is denoted as $\mathbf {\Pi }_{\text {A}}$ and it projects orthogonally onto the range space of the matrix $\text {A} \in \mathbb {R}^{m \times n}$ ($\mathcal {R}(\text {A})$). For the weighted squared norm of a vector we use the notation $||\underline{\text {y}}||^2_{\text {Q}_{\text {y} \text {y}}} = \underline{\text {y}}^T \text {Q}^{-1}_{\text {y} \text {y}} \underline{\text {y}}$. If the squared norm is with respect to (w.r.t.) the identity matrix then it is denoted $||.||^2$. A normal PDF is denoted as $\mathcal {N}(.,.)$, a chi-squared PDF as $\chi ^2(.,.)$, and a uniform PDF as $\mathcal {U}(.,.)$.            

## Review of DIA-Estimator

This section provides a concise review of the principles of *combined* parameter estimation and statistical hypothesis testing, as established in the theoretical framework of the DIA-estimator (Teunissen 2018). We start by considering a linear(ized) observation model with normally (Gaussian) distributed observables (Odijk 2017; Maaref et al. 2021; Liu et al. 2023),

2 $$\begin{aligned} \underline{\text {y}} \in \mathbb {R}^m \sim \mathcal {N}\left( \textsf{E}_{f_{\underline{\text {y}}}}\left( \underline{\text {y}} \right) , \text {Q}_{\text {y} \text {y}} \right) , \end{aligned}$$

where misspecifications could occur in the mean $\textsf{E}_{f_{\underline{\text {y}}}}\left( \underline{\text {y}} \right) \in \mathbb {R}^m$, vc-matrix $\text {Q}_{\text {y} \text {y}} \in \mathbb {R}^{m \times m}$, and/or in the assumed type of the probability distribution $f_{\underline{\text {y}}}(y)$, meaning the Gaussian assumption may not hold. In this contribution, we focus on the case of misspecifications in the mean, as these are the most common in practice (e.g., caused by outliers) (Teunissen 2017). To account for such misspecifications of the observation model, the following multiple hypothesis testing problem is formulated

3 $$\begin{aligned} \mathcal {H}_0: \textsf{E}_{f_{\underline{\text {y}}}}\left( \underline{\text {y}} \right) = \text {A} \text {x}~~~\text {vs.}~~~\mathcal {H}_{i \ne 0}: \textsf{E}_{f_{\underline{\text {y}}}}\left( \underline{\text {y}} \right) = \text {A} \text {x} + \text {C}_i \text {b}_i, \end{aligned}$$

where $\text {A} \in \mathbb {R}^{m \times n}$ is the design matrix with $\text {rank}(\text {A}) = n$, $\text {x} \in \mathbb {R}^n$ is the vector of unknown parameters, and *k*-alternative hypotheses with index $i \in \{1,...,k\}$. The outliers in the observation model are specified by the matrix $\text {C}_i \in \mathbb {R}^{m \times q_i}$, and $\text {rank}([\text {A},\text {C}_i]) = n+q_i$ with $\text {b}_i \in \mathbb {R}^{q_i}$ being the (unknown) vector containing the sizes of the outliers. The misclosure vector $\underline{\text {t}} \in \mathbb {R}^r$, which has the dimension of the redundancy $r = m - n$, can be used to build test statistics for the decision problem in (3), since it provides a measure of inconsistency between the model under $\mathcal {H}_0$ and the observables. The expressions of $\underline{\text {t}} \in \mathbb {R}^r$ and its vc-matrix $\text {Q}_{\text {t} \text {t}} \in \mathbb {R}^{r \times r}$ are Teunissen (2006)

4 $$\begin{aligned} \underline{\text {t}} = \text {B}^T \underline{\text {y}},~~~\text {Q}_{\text {t} \text {t}} = \text {B}^T \text {Q}_{\text {y} \text {y}} \text {B}, \end{aligned}$$

where $\text {B}\in \mathbb {R}^{m \times r}$ is a basis matrix of $\mathcal {R}(\text {A})^{\perp }$ (i.e., $\text {B}^T\text {A} = 0_{r \times n}$). The misclosure vector $\underline{\text {t}} \in \mathbb {R}^r$ can be used to connect the Best Linear Unbiased Estimators (BLUEs) of $\text {x} \in \mathbb {R}^n$ under the alternative hypotheses $\mathcal {H}_{i \ne 0}$’s, to the corresponding estimator under $\mathcal {H}_0$, as follows (Teunissen 2018)

5 $$\begin{aligned} \begin{bmatrix} \hat{\underline{\text {x}}}_i \\ \underline{\text {t}} \end{bmatrix} = \begin{bmatrix} \text {I}_n & -\text {L}_i \\ 0_{r \times n} & \text {I}_r \end{bmatrix} \begin{bmatrix} \hat{\underline{\text {x}}}_0 \\ \underline{\text {t}} \end{bmatrix} ,~\text {with}~ \text {L}_i = {\left\{ \begin{array}{ll} 0_{n \times r} & ,i=0\\ \text {A}^+\text {C}_i \text {C}^{+}_{\text {t}_i} & ,i\ne 0 \end{array}\right. } \end{aligned}$$

where $\hat{\underline{\text {x}}}_0 = \text {A}^+ \underline{\text {y}}$ and $\text {A}^+ = \text {Q}_{\hat{\text {x}}_0 \hat{\text {x}}_0} \text {A}^T \text {Q}_{\text {y} \text {y}}^{-1}$ is the BLUE-inverse of $\text {A} \in \mathbb {R}^{m \times n}$. The BLUE-inverse of $\text {C}_{\text {t}_i} = \text {B}^T \text {C}_i$ is $\text {C}^+_{\text {t}_i} = (\text {C}_{\text {t}_i}^T \text {Q}_{\text {t} \text {t}}^{-1}\text {C}_{\text {t}_i})^{-1}\text {C}^T_{\text {t}_i}\text {Q}_{\text {t} \text {t}}^{-1}$. The PDF of $\left[ \hat{\underline{\text {x}}}^T_i~\underline{\text {t}}^T\right] ^T$, under hypothesis $\mathcal {H}_a$ with $a\in \{0,...,k\}$, is

6 $$\begin{aligned} \mathcal {H}_a: \begin{bmatrix} \hat{\underline{\text {x}}}_i\\ \underline{\text {t}}\\ \end{bmatrix} \sim \mathcal {N} \left( \begin{bmatrix} \text {x} + \text {A}^+\text {R}_i \text {C}_a \text {b}_a\\ \text {B}^T \text {C}_a \text {b}_a \end{bmatrix},~ \begin{bmatrix} \text {Q}_{\hat{\text {x}}_0 \hat{\text {x}}_0} + \text {L}_i \text {Q}_{\text {t} \text {t}} \text {L}_i^T & -\text {L}_i \text {Q}_{\text {t} \text {t}}\\ -\text {Q}_{\text {t} \text {t}}\text {L}_i^T & \text {Q}_{\text {t} \text {t}} \end{bmatrix} \right) ~, \end{aligned}$$

where $\text {R}_i = \text {I}_m - \text {C}_i \left( \text {B}^T \text {C}_i\right) ^{+}\text {B}^T$, in which $\left( \text {B}^T \text {C}_i\right) ^{+} = \left( \text {C}^T_i \text {B}\,\text {Q}^{-1}_{\text {t} \text {t}} \text {B}^T\text {C}_i \right) ^{-1}\text {C}^T_i \text {B}\,\text {Q}^{-1}_{\text {t} \text {t}}$. The matrix $\text {R}_i$ projects along $\mathcal {R}(\text {C}_i)$ and onto $\mathcal {R}(\text {A},\text {Q}_{\text {y} \text {y}}\text {B}(\text {B}^T\text {C}_i)^{\perp })$ with $(\text {B}^T\text {C}_i)^{\perp }$ being a basis matrix of the null space of $\text {C}^T_i \text {B}$. The variance-covariance $\text {Q}_{\hat{\text {x}}_0 \hat{\text {x}}_0} = (\text {A}^T \text {Q}_{\text {y} \text {y}}^{-1} \text {A})^{-1}$ is the one of $\hat{\underline{\text {x}}}_0$. Note that for a given $\mathcal {H}_a$, the following two-cases hold for the joint PDF of $\left[ \hat{\underline{\text {x}}}^T_i~\underline{\text {t}}^T\right] ^T$,

7 $$\begin{aligned} f_{\hat{\underline{\text {x}}}_i, \underline{\text {t}}}(\textit{x},\textit{t}) {\left\{ \begin{array}{ll} = f_{\hat{\underline{\text {x}}}_0}(\textit{x})f_{\underline{\text {t}}}(\textit{t}) & ,~\text {if}~i=0\\ \ne f_{\hat{\underline{\text {x}}}_i}(\textit{x})f_{\underline{\text {t}}}(\textit{t}) & ,~\text {if}~i\ne 0, \end{array}\right. } \end{aligned}$$

which shows that, for $i\ne 0$, the BLUEs $\hat{\underline{\text {x}}}_i$ and the misclosure vector $\underline{\text {t}}$ are dependent (see an illustrative example in Section IV.1 of Ciuban et al. (2024)). By applying partitioning principles, the hypothesis testing problem in (3) can be represented in the misclosure vector space $\mathbb {R}^r$. Partitions in $\mathbb {R}^r$ can be formulated based on the subsets $\mathcal {P}_i \subset \mathbb {R}^r$, for $i \in \{0,...,k\}$, such that $\cup ^k_{i=0} \mathcal {P}_i = \mathbb {R}^r$, and $\mathcal {P}_i \cap \mathcal {P}_j = \{ 0 \}$ for $i \ne j$. The $k+1$ partitions can be defined as follows,

8 $$\begin{aligned} \begin{aligned} \mathcal {P}_0&= \left\{ \textit{t} \in \mathbb {R}^r~|~\vert \vert \textit{t} \vert \vert ^2_{\text {Q}_{\text {t} \text {t}}} \le \chi ^2_{\alpha }(r,0) \right\} , \\ \mathcal {P}_{i \ne 0}&= \left\{ \textit{t} \in \mathbb {R}^r~|~\textit{t} \notin \mathcal {P}_0,~\check{\textsf{T}}_{i}=\underset{l \in \{1,...,k\}}{\max } ~\textsf{T}_l \right\} , \end{aligned} \end{aligned}$$

where $||\underline{\text {t}}||^2_{\text {Q}_{\text {t} \text {t}}}$ is the overall model test statistic, $\chi ^2_{\alpha }(r,0)$ is the Chi-squared critical value for a level of significance $\alpha$, and $\underline{\textsf{T}}_l$ is the result of the following transformation (Teunissen 2017; Zaminpardaz and Teunissen 2023)

9 $$\begin{aligned} \underline{\textsf{T}}_l = \text {CDF}_{\chi ^2(q_l,0)}\left( \vert \vert \mathbf {\Pi }_{\text {C}_{\text {t}_l}} \underline{\text {t}} \vert \vert ^2_{\text {Q}_{\text {t} \text {t}}}\right) , \end{aligned}$$

where $\text {CDF}_{\chi ^2(q_l,0)}(.)$ is the cumulative distribution function (CDF) of $\chi ^2(q_l,0)$, $\vert \vert \mathbf {\Pi }_{\text {C}_{\text {t}_l}} \underline{\text {t}} \vert \vert ^2_{\text {Q}_{\text {t} \text {t}}} \overset{\mathcal {H}_0}{\sim } \chi ^2(q_l,0)$, $\mathbf {\Pi }_{\text {C}_{\text {t}_l}} = \text {C}_{\text {t}_l} \text {C}^+_{\text {t}_l}$ projects onto $\mathcal {R}(\text {C}_{\text {t}_l})$, and $\text {C}_{\text {t}_l} = \text {B}^T \text {C}_l$ while $\text {C}^+_{\text {t}_l} = (\text {C}_{\text {t}_l}^T \text {Q}_{\text {t} \text {t}}^{-1}\text {C}_{\text {t}_l})^{-1}\text {C}^T_{\text {t}_l}\text {Q}_{\text {t} \text {t}}^{-1}$, and $\underline{\textsf{T}}_l \overset{\mathcal {H}_0}{\sim } \mathcal {U}(0,1)$. Note that the evaluation of $\text {CDF}_{\chi ^2(q_l,0)}(.)$ must be done *k*-times, which can be computationally expensive for a larger number of alternative hypothesis. To mitigate this, one can: (i) group the alternative hypotheses by their common dimension $q_{l}$, and denote the number of resulting groups as *g*; for a simple example with $m = 4$, $n=1$, $r=3$, and $k=10$, we have four one-dimensional outliers, one per observation $q_{l \in \{1,...,4\}} = 1$ and six two-dimensional ones $q_{l \in \{5,...,10\}} = 2$, and thus $g = 2$; (ii) within each group $\mathcal {G}$, compute $\underset{l \in \mathcal {G}}{\max } ~\vert \vert \mathbf {\Pi }_{\text {C}_{\text {t}_l}} \underline{\text {t}} \vert \vert ^2_{\text {Q}_{\text {t} \text {t}}}$; (iii) evaluate the corresponding CDF at each of these maximum values; (iv) select the maximum among these *g* transformed CDF values. This procedure reduces the evaluation of the CDF from *k*-times to *g*-times.

**Fig. 1:**
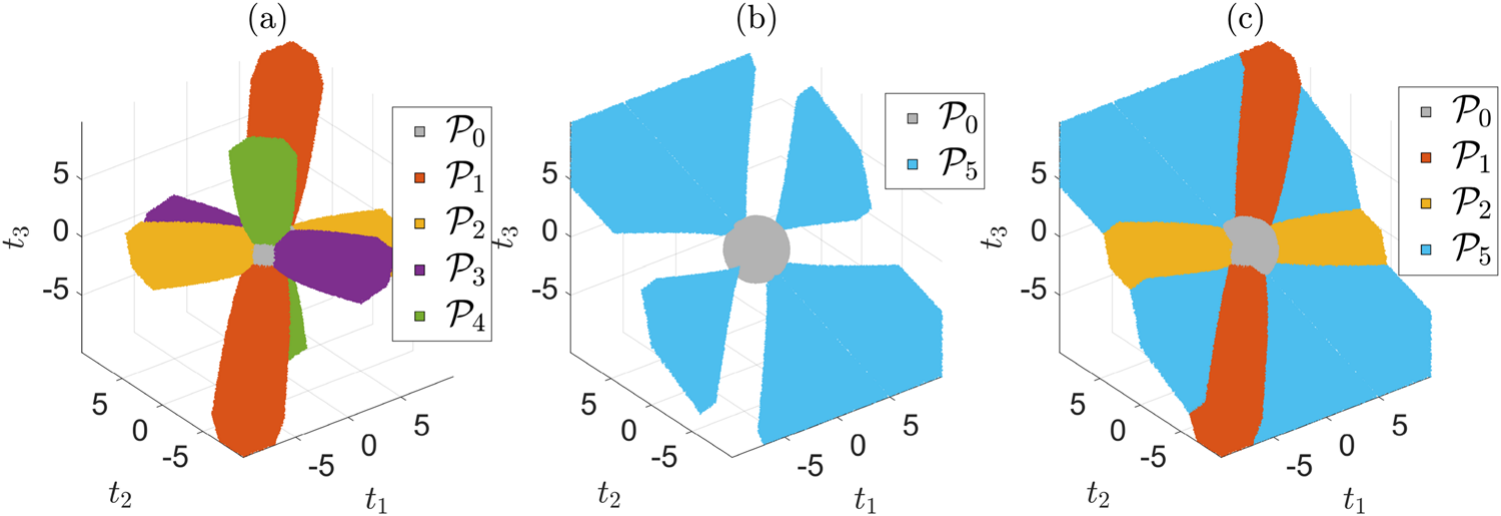
Partition of $\mathbb {R}^{r=3}$ when $\text {A}=[1~1~1~1]^T \in \mathbb {R}^{4 \times 1},~\text {x}\in \mathbb {R},~\text {Q}_{\text {y} \text {y}} = \text {I}_4$, $\text {c}_i \in \mathbb {R}^{4 \times (q_i = 1)}$ models one-dimensional outliers for $i =\{1,..,4\}$ (e.g., $$\text {c}_1 = \begin{bmatrix} 1&0&0&0 \end{bmatrix}^T$$, and $\text {C}_i \in \mathbb {R}^{4 \times (q_i = 2)}$ model two dimensional outliers for $i =\{5,...,10\}$ (e.g., $$\text {C}_5 = \begin{bmatrix} 1 & 0 & 0 & 0 \\ 0 & 1 & 0 & 0 \end{bmatrix}^T = \begin{bmatrix} \text {c}^T_1 \\ \text {c}^T_2 \end{bmatrix}^T$$). Partioning is done according to (8) for all $k+1$ hypotheses, with $k=10$. As examples: (a) shows partitions $\mathcal {P}_0$ and $\mathcal {P}_{i \ne 0}$ for $i \in \{1,...,4\}$ in colors, while the white space represents $\mathbb {R}^{r=3}\backslash (\cup ^4_{i=0} \mathcal {P}_i)$; (b) shows partitions $\mathcal {P}_0$ and $\mathcal {P}_{5}$ while the white space represents $\mathbb {R}^{r=3}\backslash (\cup _{i \in \{0,5\}} \mathcal {P}_i)$; (c) partitions $\mathcal {P}_0$ and $\mathcal {P}_{i \ne 0}$ for $i \in \{1,2,5\}$ while the white space represents $\mathbb {R}^{r=3}\backslash (\cup _{i \in \{0,1,2,5\}} \mathcal {P}_i)$

The role of the partitions in (8) is such that a hypothesis $\mathcal {H}_i$, for $i \in \{0,...,k\}$, is selected as the most likely one if and only if $\underline{\text {t}} \in \mathcal {P}_i$. Figure 1 shows the partitions (volumes) obtained when considering one and two-dimensional model outliers for a simple example.

Note that an undecided region $\Omega \subset \mathbb {R}^r$ can be included to accommodate situations when it would be difficult to discriminate between hypotheses or when not all possible outliers can be anticipated. In this contribution, we consider that always an estimator is selected (i.e., no $\Omega \subset \mathbb {R}^r$ is included). The decision outcome in statistical hypothesis testing is determined by where the misclosure vector $\underline{\text {t}}$ lands in  $\mathbb {R}^r$ with partitions $\mathcal {P}_i \subset \mathbb {R}^r$, for $i \in \{0,..,k\}$, cf. (8). Under $\mathcal {H}_0$, the decision results are: (i) Correct Acceptance (CA) of $\mathcal {H}_0$ when $\underline{\text {t}} \in \mathcal {P}_0$, and (ii) False Alarm (FA) when $\underline{\text {t}} \notin \mathcal {P}_0$, or specifically the FA per alternative hypothesis (i.e., $\text {FA}_i$) when $\underline{\text {t}} \in \mathcal {P}_i$ for $i>0$. The probabilities of these decisions are given by

10 $$\begin{aligned} \textsf{P}_{\text {CA}}= \textsf{P}\left( \underline{\text {t}} \in \mathcal {P}_0|\mathcal {H}_0 \right) ,~~ \textsf{P}_{\text {FA}}= \sum ^k_{i=1} \underbrace{\textsf{P}\left( \underline{\text {t}} \in \mathcal {P}_i|\mathcal {H}_0 \right) }_{\textsf{P}_{\text {FA}_i}}, \end{aligned}$$

where $\textsf{P}_{\text {FA}} = \alpha$ is the level of significance and $\textsf{P}_{\text {CA}} + \textsf{P}_{\text {FA}} = 1$. Similarly, under $\mathcal {H}_i$, for $i > 0$, the outcomes of the decisions are: (i) missed detection (MD) when $\underline{\text {t}} \in \mathcal {P}_0$, (ii) Correct Identification (CI) when $\underline{\text {t}} \in \mathcal {P}_i$, and (iii) Wrong Identification (WI) $\underline{\text {t}} \in \mathcal {P}_j$ for $j\notin \{0,i\}$. Their probabilities are

11 $$\begin{aligned} \begin{aligned}&\textsf{P}_{\text {MD}_i} = \textsf{P}\left( \underline{\text {t}} \in \mathcal {P}_0|\mathcal {H}_i \right) ,~~\textsf{P}_{\text {CI}_i} = \textsf{P}\left( \underline{\text {t}} \in \mathcal {P}_i|\mathcal {H}_i \right) ,\\&\textsf{P}_{\text {WI}} = \sum ^k_{j \ne 0,i} \underbrace{\textsf{P}\left( \underline{\text {t}} \in \mathcal {P}_j|\mathcal {H}_i \right) }_{\textsf{P}_{\text {WI}_j}}, \end{aligned} \end{aligned}$$

where the decision outcome of Correct Detection (CD) is given by $\textsf{P}_{\text {CD}_i} = \textsf{P}_{\text {CI}_i} + \textsf{P}_{\text {WI}}$ satisfying $\textsf{P}_{\text {MD}_i} + \textsf{P}_{\text {CD}_i} = 1$. The probabilities in (11) depend on the unknown vector $\text {b}_i \in \mathbb {R}^{q_i}$ since $f_{\underline{\text {t}}}(t|\mathcal {H}_i) = \mathcal {N}\left( \text {B}^T \text {C}_i \text {b}_i, \text {Q}_{\text {t} \text {t}}\right)$.

The Detection ($\texttt{D}$) Identification ($\texttt{I}$) and Adaptation ($\texttt{A}$) statistical testing procedure is then

12 $$\begin{aligned} {\left\{ \begin{array}{ll} \text {if}~\underline{\text {t}} \in \mathcal {P}_0~{\text {(no}~\texttt{D})} \rightarrow ~\text {output}~\hat{\underline{\text {x}}}_0,\\ \text {if}~\underline{\text {t}} \notin \mathcal {P}_0~{(\texttt{D})} \rightarrow ~\underline{\text {t}} \in \mathcal {P}_{i \ne 0}~{(\texttt{I})} \rightarrow ~\text {output}~\hat{\underline{\text {x}}}_i~{(\texttt{A})},\\ \end{array}\right. } \end{aligned}$$

where $\hat{\underline{\text {x}}}_0 \in \mathbb {R}^n$ and $\hat{\underline{\text {x}}}_i \in \mathbb {R}^n$ are the Best Linear Unbiased Estimators (BLUEs) of $\text {x} \in \mathbb {R}^n$ under $\mathcal {H}_0$ and $\mathcal {H}_{i \ne 0}$. The estimator that captures the procedure in (12) is the DIA-estimator (Teunissen 2018)

13 $$\begin{aligned} \overline{\underline{\text {x}}} = \sum ^k_{i=0} \hat{\underline{\text {x}}}_i\, p_i(\underline{\text {t}}) = \hat{\underline{\text {x}}}_0 - \sum ^k_{i=1} \text {L}_i\,\underline{\text {t}}\, p_i(\underline{\text {t}}), \end{aligned}$$

where the indicator function $p_i(\underline{\text {t}}) = 1$ if $\underline{\text {t}} \in \mathcal {P}_i$ and $p_i(\underline{\text {t}}) = 0$ otherwise. The uncertainty of parameter estimation is carried by the BLUEs $\hat{\underline{\text {x}}}_i$ and uncertainty of statistical testing is carried by $p_i(\underline{\text {t}})$. Note that $p_i(\underline{\text {t}})$ is a nonlinear function of $\underline{\text {t}}$, which causes the PDF of the DIA-estimator $\underline{\overline{\text {x}}}$ to be *multimodal*, even though the individual PDFs of the BLUEs $\hat{\underline{\text {x}}}_i$ are Gaussian. The PDF of $\overline{\underline{\text {x}}} \in \mathbb {R}^n$ follows from Theorem 1 in Teunissen (2018)

14 $$\begin{aligned} f_{\overline{\underline{\text {x}} }}\left( \textit{x}\right) = \sum _{i=0}^{k} \int _{\mathcal {P}_i} f_{\hat{\underline{\text {x}}}_i,\underline{\text {t}}}\left( \textit{x},\textit{t} \right) d\textit{t}~. \end{aligned}$$

The conditional components of $f_{\overline{\underline{\text {x}} }}\left( \textit{x}\right)$ on the testing decisions under $\mathcal {H}_0$ (i.e., $\text {CA}$ and $\text {FA}_i$) can be obtained from $f_{\overline{\underline{\text {x}} }}\left( \textit{x} |\mathcal {H}_0\right)$. Those corresponding to the testing decisions under $\mathcal {H}_i$ (i.e., $\text {MD}_i$, $\text {CI}_i, \text {WI}_j$) from $f_{\overline{\underline{\text {x}} }}\left( \textit{x} |\mathcal {H}_i\right)$ for $i\ne 0$. It is the DIA-estimator’s PDF in (14) that is used in the formulation of the probability of positioning failure. We note that one may only be interested in specific components of the parameter vector $\text {x} \in \mathbb {R}^n$, such as the 1D, 2D, or 3D position. The desired position components can be obtained through the linear transformation $\text {h} = \text {H}^T \text {x}$ with an appropriately chosen $\text {H} \in \mathbb {R}^{n \times p}$ where $p < n$. Further developments in this paper are done in terms of $\text {x} \in \mathbb {R}^n$, however a similar approach would apply also for $\text {h} \in \mathbb {R}^p$.

## Probability of positioning failure and its components

**Fig. 2:**
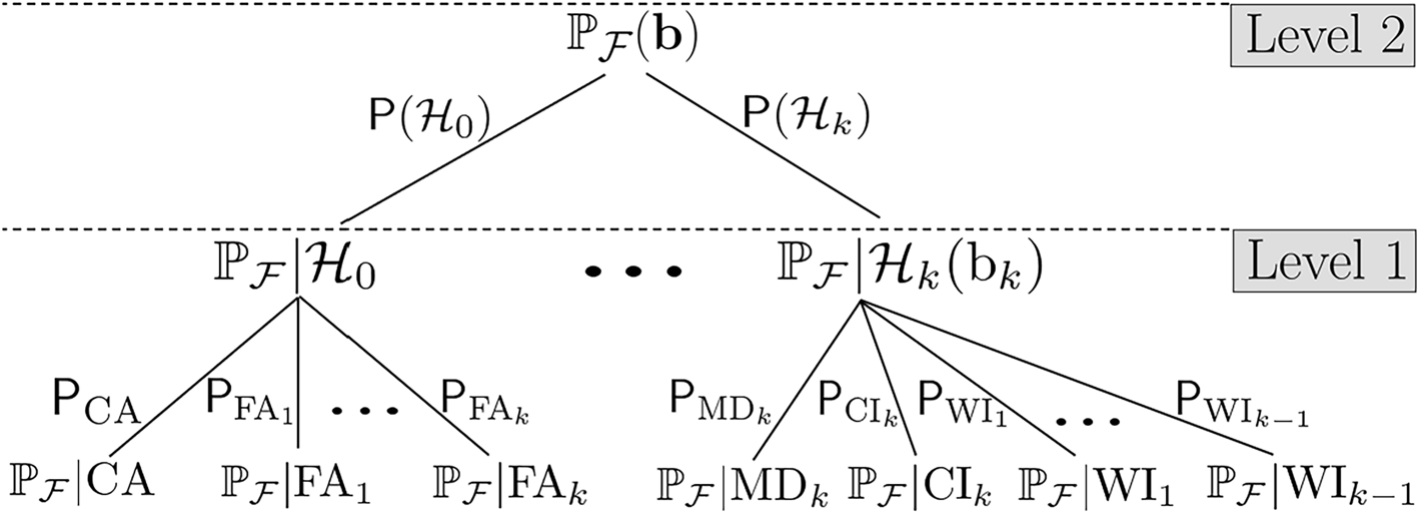
Representation of $\mathbb {P}_{\mathcal {F}}(\textbf{b})$ as a ’failure-tree’. Level 1 corresponds to (19) and Level 2 to (16)

The *positioning failure* event is defined as $\mathcal {F} = \overline{\underline{\text {x}}} \in \mathcal {B}^c$, according to page 15 of Special Committee 159, R.T.C.A. (2020), where $\mathcal {B} \subset \mathbb {R}^{n}$ denotes the safety region, and its complement $\mathcal {B}^c = \mathbb {R}^n \setminus \mathcal {B}$ represents the failure region. We now proceed to formulate the probability of positioning failure

15 $$\begin{aligned} \mathbb {P}_{\mathcal {F}} (\textbf{b}) = \int _{\mathcal {B}^c} f_{\overline{\underline{\text {x}}}}(x)d x, \end{aligned}$$

where $\textbf{b} = \{\text {b}_1 \in \mathbb {R}^{q_1},\text {b}_2 \in \mathbb {R}^{q_2},...,\text {b}_k \in \mathbb {R}^{q_k}\}$ represents the vectors of the outliers in the observation model under the *k*-alternative hypotheses. In general, the dimensions of the model outliers may differ, i.e., $q_i \ne q_j$ for $i \ne j$. The next step is to decompose (15) via the rule of total probability

16 $$\begin{aligned} \begin{aligned} \mathbb {P}_{\mathcal {F}} (\textbf{b}) = \textsf{P}(\mathcal {H}_0)&\underbrace{\int _{\mathcal {B}^c} f_{\overline{\underline{\text {x}}}}(\textit{x}|\mathcal {H}_0) dx}_{\mathbb {P}_{\mathcal {F}}|\mathcal {H}_0 } + \sum ^k_{i=1}\textsf{P}(\mathcal {H}_i) \underbrace{\int _{\mathcal {B}^c} f_{\overline{\underline{\text {x}}}}(\textit{x}|\mathcal {H}_i) dx}_{\mathbb {P}_{\mathcal {F}}|\mathcal {H}_i(\text {b}_i)}, \end{aligned} \end{aligned}$$

where $\textsf{P}(\mathcal {H}_0)$ and $\textsf{P}(\mathcal {H}_i)$ are the apriori probability of occurrence of the hypotheses. A further expansion of (16), based on (14), gives

17 $$\begin{aligned} \begin{aligned} \mathbb {P}_{\mathcal {F}} (\textbf{b}) = \textsf{P}&(\mathcal {H}_0) \left( \sum ^k_{j=0} \int _{\mathcal {B}^c} \int _{\mathcal {P}_j} f_{\hat{\underline{\text {x}}}_j,\underline{\text {t}}}\left( x,t|\mathcal {H}_0\right) dt\,dx \right) + \\&~~~~~~~~~~~~~~~~\sum _{i=1}^{k} \textsf{P}(\mathcal {H}_i) \left( \sum ^k_{j=0} \int _{\mathcal {B}^c} \int _{\mathcal {P}_j} f_{\hat{\underline{\text {x}}}_j,\underline{\text {t}}}\left( x,t|\mathcal {H}_i \right) dt\,dx \right) , \end{aligned} \end{aligned}$$

which can then be expressed in terms of expected values w.r.t. the joint PDFs $f_{\hat{\underline{\text {x}}}_j,\underline{\text {t}}}\left( \textit{x},\textit{t} \right)$

18 $$\begin{aligned} \begin{aligned} \mathbb {P}_{\mathcal {F}} (\textbf{b}) = \textsf{P}(\mathcal {H}_0)&\left( \sum ^k_{j=0} \textsf{E}_{f_{\hat{\underline{\text {x}}}_j,\underline{\text {t}}}}\left( 1\!\!1_j(\underline{\text {x}}, \underline{\text {t}} )|\mathcal {H}_0 \right) \right) \\&+ \sum ^k_{i=1} \textsf{P}(\mathcal {H}_i)\left( \sum ^k_{j=0} \textsf{E}_{f_{\hat{\underline{\text {x}}}_j,\underline{\text {t}}}}\left( 1\!\!1_j(\underline{\text {x}}, \underline{\text {t}} )|\mathcal {H}_i \right) \right) , \end{aligned} \end{aligned}$$

with the joint indicator function $1\!\!1_j(\underline{\text {x}}, \underline{\text {t}}) = 1$ if $[\underline{\text {x}}^T~\underline{\text {t}}^T]^T \in \left( \mathcal {B}^c \cap \mathcal {P}_j \right)$, and 0 otherwise. The two summations of joint probabilities in parentheses in (18) are rewritten below, explicitly incorporating the statistical testing decisions from (10)-(11) into the notation,

19 $$\begin{aligned} \begin{aligned} \sum ^k_{j=0} \textsf{E}_{f_{\hat{\underline{\text {x}}}_j,\underline{\text {t}}}}\left( 1\!\!1_j(\underline{\text {x}}, \underline{\text {t}} )|\mathcal {H}_0 \right)&= \mathbb {P}_{\mathcal {F}}|\text {CA}\,\textsf{P}_{\text {CA}} +\sum _{j=1}^{k}\mathbb {P}_{\mathcal {F}}|\text {FA}_j\,\textsf{P}_{\text {FA}_j}, \\ \sum ^k_{j=0} \textsf{E}_{f_{\hat{\underline{\text {x}}}_j,\underline{\text {t}}}}\left( 1\!\!1_j(\underline{\text {x}}, \underline{\text {t}} )|\mathcal {H}_i \right)&= \mathbb {P}_{\mathcal {F}}|\text {MD}_i\,\textsf{P}_{\text {MD}_i}+ \mathbb {P}_{\mathcal {F}}|\text {CI}_i\,\textsf{P}_{\text {CI}_i} + \sum _{j \ne 0,i}^{k}\mathbb {P}_{\mathcal {F}}|\text {WI}_j\,\textsf{P}_{\text {WI}_{j}}~. \end{aligned} \end{aligned}$$

where $\mathbb {P}_{\mathcal {F}}|\mathcal {E}$ is the probability of positioning failure conditioned on the testing decision $\mathcal {E}\in \{\text {CA},\,\text {FA}_j,\,\text {MD}_i,\,\text {CI}_i,\,\text {WI}_{j}\}$. These decompositions are schematically illustrated as a ’failure-tree’ in Fig. 2, where Level 1 corresponds to (19) and Level 2 corresponds to (16). To obtain the Level 1 and Level 2 components depicted in Fig. 2, one can resort to Monte Carlo simulation to approximate the expected values in (18). However, if these components are on the order of $10^{-4}$ or less, the variance of the results of the standard Monte Carlo simulation may be too high or the probability may not be even computable (Rubinstein and Kroese 2008). We have addressed these challenges in recent work (Ciuban et al. 2025b), where we developed a method that enables the construction of the ’failure-tree’ in Fig. 2 by leveraging principles from rare event simulation–an approach also applied in this contribution. We give the three main steps of the proposed method below:

1. Transform the joint vector $[\underline{\hat{\text {x}}}_j^T~\underline{\text {t}}^T]^T$ to have an identity covariance matrix, and apply this change of variables to re-express the expectations in (18).
2. Reformulate these expectations using proposal PDFs based on the Importance Sampling principle (Kahn and Marshall 1953), selecting PDFs with higher density over $\mathcal {B}^c \cap \mathcal {P}_j$, as determined via the Cross-Entropy Method (Rubinstein 1999).
3. Compute the Level 1 and Level 2 components of $\mathbb {P}_{\mathcal {F}} (\textbf{b})$ by drawing i.i.d. samples from the proposal PDFs, and assess simulation uncertainty through multiple independent runs (e.g., 50 repetitions (Morio and Balesdent 2016)).

After completing the computations, the maximum of (15) can be evaluated and compared against an application-specific requirement to determine whether the requirement is satisfied (e.g., (Ciuban et al. 2024, 2025a, 2025b))

20 $$\begin{aligned} \underset{\textbf{b}}{\max }\,\mathbb {P}_{\mathcal {F}} (\textbf{b}) = \textsf{P}(\mathcal {H}_0)\mathbb {P}_{\mathcal {F}}|\mathcal {H}_0 + \underset{\text {b}_1,...,\text {b}_k}{\max } \sum ^k_{i=1} \textsf{P}(\mathcal {H}_i) \mathbb {P}_{\mathcal {F}}|\mathcal {H}_i(\text {b}_i). \end{aligned}$$

## Positioning safety analysis for a UAV under 2D simultaneous measurement outliers

As an example, we present a positioning safety analysis for a UAV under multiple simultaneous outliers in the observations. The analysis is carried out for two scenarios using the principles from Sects. 2 and 3. The first scenario considers a GPS satellite geometry as observed by a receiver at a snapshot of time, enabling detailed analysis of the conditional components of the DIA-estimator’s PDF relative to the safety region shape, as well as the computed components of the probability of positioning failure. In the second scenario we consider a 24-hours (on May 24, 2024) evolution of GPS satellites moving over an airspace region in The Netherlands for which authorization can be obtained for UAV operations (Latitude: 51.74368 [deg], Longitude 3.79322 [deg], Altitude: 0.0 [m], based on EASA online), while the components of the probability of positioning failure are computed at 5 min intervals. The selected airspace region has open-sky conditions while within it, the UAV is assumed to operate at a constant altitude of 45 m above ground level ensuring compliance with height regulations. In both scenarios we are using real GPS satellite orbits provided by the International GNSS Service (IGS) (Johnston et al. 2017). The positioning model for the UAV under nominal conditions (i.e., under $\mathcal {H}_0$) is selected based on (i) the TSO certification standards for GPS-based UAV positioning (FAA 2017; Cozzens 2021), and (ii) preliminary requirements for rotary-wing drones operating at SAIL level 4, as outlined in EUSPA (2023). Therefore, the linearized positioning model is based on GPS pseudoranges on single-frequency (L1 at 1575.42 MHz) resulting in

21 $$\begin{aligned} \mathcal {H}_0:\textsf{E}_{f_{\underline{\text {y}}}}\left( \underline{\text {y}} \right) = \underbrace{ \begin{bmatrix}\text {G}&\textbf{u}_m \end{bmatrix}}_{\text {A}} \underbrace{\begin{bmatrix} \Delta \textsf{p} \\ \texttt{c} \Delta \textsf{t} \end{bmatrix}}_{\Delta \text {x}},~~~~\text {Q}_{\text {y} \text {y}}=\sigma ^2_{\text {y}} \text {W}^{-1}, \end{aligned}$$

with $\underline{\text {y}} \sim \mathcal {N}\left( \text {A}\, \Delta \text {x}, \text {Q}_{\text {y} \text {y}} \right)$, where the design matrix $\text {A} \in \mathbb {R}^{m \times 4}$ is of $\text {rank}(\text {A}) = 4$ (full rank) with $\text {G} \in \mathbb {R}^{m \times 3}$ the matrix which rows contain the unit direction vectors (with the minus sign included) between the unknown position of the UAV’s GPS receiver and the observed GPS satellites, in a local East-North-Up (ENU) coordinate system, and $\textbf{u}_m \in \mathbb {R}^m$ is a vector of ones (Odijk 2017). The parameter vector of unknowns $\Delta \text {x} \in \mathbb {R}^4$ contains the UAV’s GPS receiver ENU coordinates increments $\Delta \text {p} \in \mathbb {R}^3$ and the receiver clock bias $\Delta \text {t} \in \mathbb {R}$ while $\texttt{c}$ is the speed of light in a vacuum. The redundancy under $\mathcal {H}_0$ is $r = m - 4$. The estimate $\hat{\text {x}}$ (which includes the 3D full position vector and receiver clock bias) is obtained from a Gauss-Newton iteration scheme once the stop criterion is met for $\Delta \hat{\text {x}}$ (Teunissen 1990). The variance-covariance matrix $\text {Q}_{ \text {y} \text {y} } \in \mathbb {R}^{m \times m}$ is diagonal where $\text {W} = \text {diag}[\omega _{1},...,\omega _{m}] \in \mathbb {R}^{m \times m}$ is the weight matrix whose components are the elevation-dependent weighting functions based on Euler and Goad (1991): $\omega _{s} = 1/(a_0 + a_1 \exp (-\text {Elev}_s/E_0)^2)$ for $s \in \{1,...,m\}$ with $a_0$, $a_1$, $E_0$ being the model coefficients and $\text {Elev}_s$ being the elevation of satellite *s* in degrees. For both scenarios, we set $a_0=1.4$, $a_1=8$, $E_0=20$, as in Euler and Goad (1991), and $\sigma _{\text {y}} = 0.7~[\text {m}]$, which results in an average horizontal positioning precision of 3–4 m (95$\%$ circular probability radius), and an average vertical positioning precision of approximately 6 m (95$\%$ interval length), considering variations in satellite geometry over a 24 h period. These positioning precisions are representative of certified GPS receivers used in UAVs (e.g., (uAvionix online)). An elevation cut-off angle of $10^{\circ }$ is applied, which excludes GPS satellites observed below this threshold from the positioning model.

### GPS satellite geometry at a snapshot of time 

**Fig. 3:**
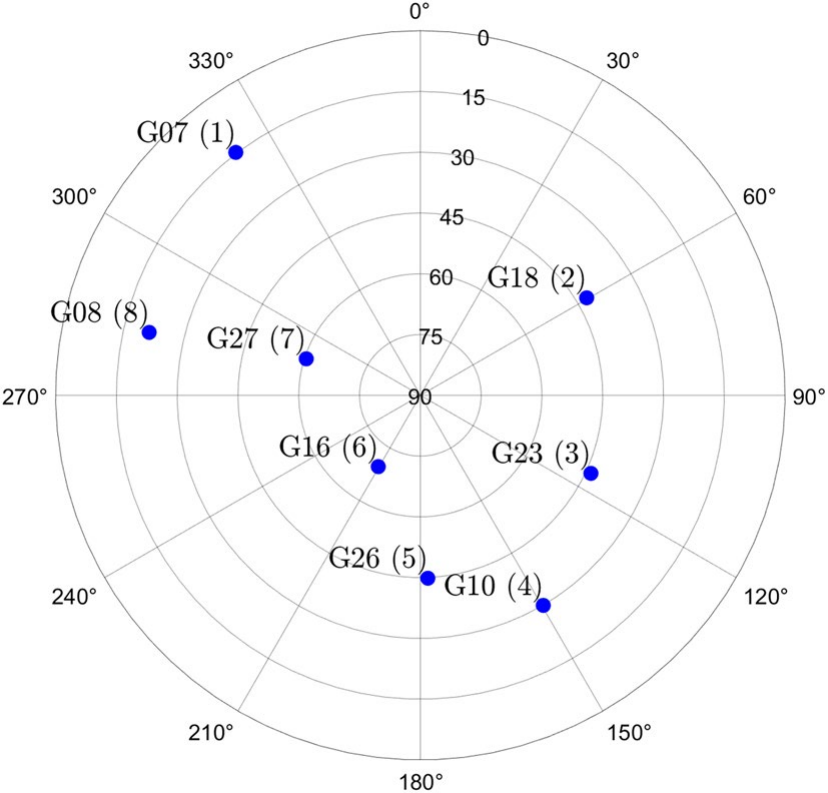
Skyplot view of the UAV GPS receiver-satellite geometry at an example location, with open-sky conditions, in Delft, The Netherlands (Latitude: 52 [deg], Longitude: 4 [deg], Altitude 0.0 [m]). GPS satellites IDs are renumbered clock-wise starting from G07 (1) and ending with G08 (8)

**Fig. 4:**
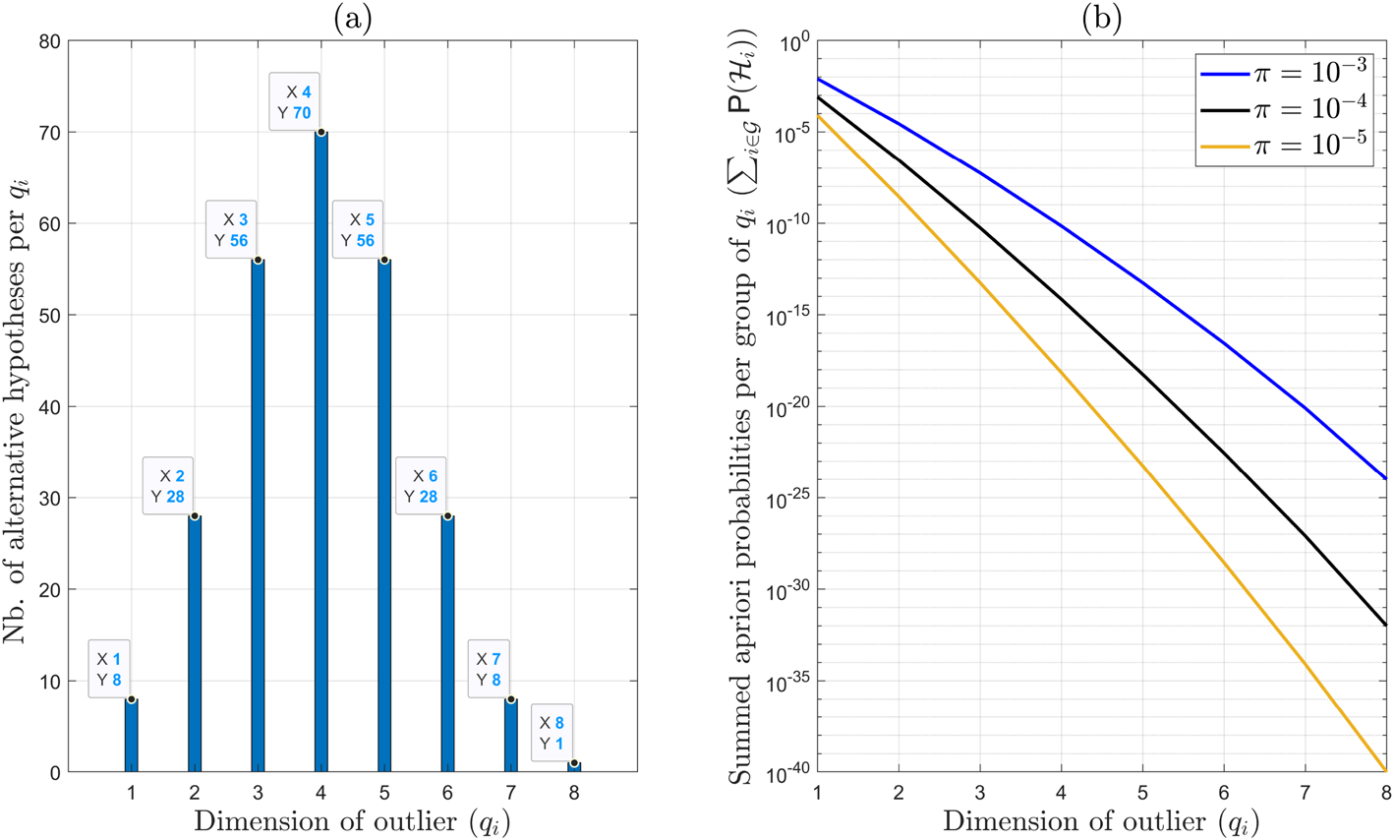
(a) Total number of alternative hypotheses corresponding to each value of $q_i$ in (22); (b) Summed apriori probabilities of alternative hypotheses $\mathcal {H}_i$ per group $\mathcal {G}$ of $q_i$ based on (23). For example, when the probability of one-dimensional outlier is $\pi = 10^{-3}$ (blue curve): $q_i = 1$ and $\sum ^{8}_{i=1} \textsf{P}(\mathcal {H}_i) = 794.41677 \cdot 10^{-5}$, $q_i = 2$ and $\sum ^{36}_{i=9} \textsf{P}(\mathcal {H}_i) = 2.78324 \cdot 10^{-5}$. Other curves correspond to the cases when $\pi = 10^{-4}$ and $\pi = 10^{-5}$

The skyplot in Fig. 3 shows the positions of eight (8) GPS satellites, at a snapshot of time, as observed by the UAV’s GPS receiver. The corresponding dimensions of the vectors in the positioning model in (21) are: $m = 8$, $n=4$, and $r =4$. Next, we motivate the consideration of one- and two-dimensional simultaneous outliers in the pseudorange observations, and provide the resulting number of alternative hypotheses for the testing procedure. Let us begin by determining the total number of possible one-dimensional and multi-dimensional pseudorange outliers among the *m*-observations (i.e., alternative hypotheses), based on the positioning model in (21) and the GPS satellite geometry shown in Fig. 3. This number can be computed as follows (Lehmann and Lösler 2016; Koch 2017; Imparato 2016)

22 $$\begin{aligned} k'= \sum ^{m=8}_{q_i=1} \left( {\begin{array}{c}m\\ q_i\end{array}}\right) = 255, \end{aligned}$$

where the binomial coefficient $$\left( {\begin{array}{c}m\\ q_i\end{array}}\right) $$ gives the number of possible combinations of $q_i$ outliers in *m*-observations. While not all of these alternative hypotheses can really be included in the testing procedure due to redundancy and separability limitations, the total count illustrates the full set of potential simultaneous outliers that could occur in practice (e.g., up to eight simultaneous outliers in the event of a constellation failure). The number resulting for each $q_i \in \{1,...,8\}$ is shown in Fig. 4(a). In the next step, we model the probability of occurrence of an alternative hypothesis $\textsf{P}(\mathcal {H}_i)$ corresponding to a $q_i$-dimensional outlier. Under open-sky conditions for the GPS receiver, it is reasonable to model $\textsf{P}(\mathcal {H}_i)$ as a decreasing function of $q_i$. Denoting $\pi$ as the probability of the occurrence of one-dimensional outlier, and assuming that outlier occurrences are independent, then $\textsf{P}(\mathcal {H}_i)$ can be modelled as follows (Blanch 2015; Working Group C 2016; Liu et al. 2022)

23 $$\begin{aligned} \textsf{P}(\mathcal {H}_i) = \prod _{\begin{array}{c} a = 1 \\ 1\!\!1_{\mathcal {H}_i}(a) = 1 \end{array}}^{m} \pi \prod _{\begin{array}{c} a = 1 \\ 1\!\!1_{\mathcal {H}_i}(a) = 0 \end{array}}^{m} (1 - \pi )= \prod ^{m}_{a=1} \pi ^{1\!\!1_{\mathcal {H}_i}(a) }(1-\pi )^{\left[ 1-1\!\!1_{\mathcal {H}_i}(a)\right] }\,, \end{aligned}$$

where $1\!\!1_{\mathcal {H}_i}(a) =1$ if the $a^{\text {th}}$ observation contains an outlier under hypothesis $\mathcal {H}_i$, and 0 otherwise. Note that $\sum ^m_{a=1} 1\!\!1_{\mathcal {H}_i}(a) = q_i$ equals the number of outliers under $\mathcal {H}_i$ and also that (23) yields the probability of the null hypothesis, $\textsf{P}(\mathcal {H}_0) = (1 - \pi )^{m}$, corresponding to $q_0 = 0$. The probabilities satisfy the condition $\sum ^{k'}_{i=0} \textsf{P}(\mathcal {H}_i) = 1$. Furthermore, (23) can be re-expressed in a simpler form

24 $$\begin{aligned} \textsf{P}(\mathcal {H}_i) = \pi ^{q_i}(1 - \pi )^{m - q_i}. \end{aligned}$$

The model from (24), along with the specified positioning safety requirements (e.g., SAIL 4), can be used to determine the maximum dimension $q_i$, which also depends on the redundancy, to be included in the DIA procedure (see (12)). For example, let us consider the situation when the redundancy $r=4$ and $\pi = 10^{-3}$. In this case, we obtain (see also Fig. 4(b))

25 $$\begin{aligned} \begin{aligned} 1 - \textsf{P}(\mathcal {H}_0)&= \underbrace{\sum ^{8}_{i=1} \textsf{P}(\mathcal {H}_i)}_{q_i = 1} + \underbrace{\sum ^{36}_{i=9} \textsf{P}(\mathcal {H}_i)}_{q_i = 2} + \underbrace{\sum ^{255}_{i=37} \textsf{P}(\mathcal {H}_i)}_{q_i \in \{3,...,8\}} \\&=794.41677 \cdot 10^{-5} + 2.78324 \cdot 10^{-5} + 0.00557 \cdot 10^{-5}\,, \end{aligned} \end{aligned}$$

which indicates that, given the SAIL 4 requirement for $\mathbb {P}_{\mathcal {F}}(\textbf{b})$ to be on the order of $10^{-5}$, alternative hypotheses with dimensions $q_i > 2$ can be excluded from the testing procedure, as their contribution to the overall probability $\mathbb {P}_{\mathcal {F}}(\textbf{b})$ is negligible and also the redundancy does not allow for the identification of outliers with dimensions $q_i > 2$. Therefore, the misclosure space $\mathbb {R}^{r=4}$ is partitioned, according to (8), while accounting for one- and two-dimensional $q_i \in \{1,2\}$ resulting in 36 alternative hypotheses ($k=36$). For the positioning safety analysis, we focus on the horizontal component (a similar approach can be applied to the vertical component). The corresponding DIA estimator for the horizontal position of the UAV is

26 $$\begin{aligned} \overline{\underline{\text {h}}} = \text {H}^T \overline{\underline{\text {x}}}, \end{aligned}$$

where $\overline{\underline{\text {x}}} = \sum ^{k=36}_{i=0} \hat{\underline{\text {x}}}_i\, p_i(\underline{\text {t}})$ cf. (13), $$\text {H}^T = \begin{bmatrix} \text {I}_2&0_{2 \times 2} \end{bmatrix}$$. The horizontal safety-region $\mathcal {B}_{\text {h}} \subset \mathbb {R}^2$, around the true position, is defined as follows

27 $$\begin{aligned} \mathcal {B}_{\text {h}} = \{ h \in \mathbb {R}^2~|~\vert \vert h - \text {h}_{\text {true}} \vert \vert \le \text {HAL} \}, \end{aligned}$$

where the Horizontal Alert Limit (HAL) is set to 11 m, in line with SAIL 4 for en-route operations (EUSPA 2023). The probability of the UAVs horizontal positioning failure is upperbounded (accounting for (25) and for the SAIL 4 requirement), as follows

28 $$\mathbb{P}_{\mathcal{F}_{\mathrm{h}}} (\mathbf{b}) \leq \underbrace{\left( \mathsf{P}(\mathcal{H}_0) \underbrace{\int_{\mathcal{B}^c_{\mathrm{h}}} f_{\overline{\underline{\mathrm{h}}}}(h|\mathcal{H}_0)dh}_{\mathbb{P}_{\mathcal{F}_{\mathrm{h}}}|\mathcal{H}_0} + \sum^{k=36}_{i=1}\mathsf{P}(\mathcal{H}_i) \underbrace{\int_{\mathcal{B}^c_{\mathrm{h}}} f_{\overline{\underline{\mathrm{h}}}}(h|\mathcal{H}_i)dh}_{\mathbb{P}_{\mathcal{F}_{\mathrm{h}}}|\mathcal{H}_i(\mathrm{b}_i)} + \sum^{k'=255}_{i=37}\mathsf{P}(\mathcal{H}_i)\right)}_{\breve{\mathbb{P}}_{\mathcal{F}_{\mathrm{h}}} (\breve{\mathbf{b}})},$$

where the term $\sum ^{k'=255}_{i=37}\textsf{P}(\mathcal {H}_i)$ accounts for the hypotheses which were ’excluded’ from the testing procedure (see (25)) and $\breve{\textbf{b}}=\{\text {b}_1,...,\text {b}_{36}\} \subset \textbf{b}$. The objective is to base the positioning safety analysis in the following subsections on the results obtained from computing $\breve{\mathbb {P}}_{\mathcal {F}_{\text {h}}} (\breve{\textbf{b}})$.    

#### Results under $\mathcal {H}_0$

**Fig. 5:**
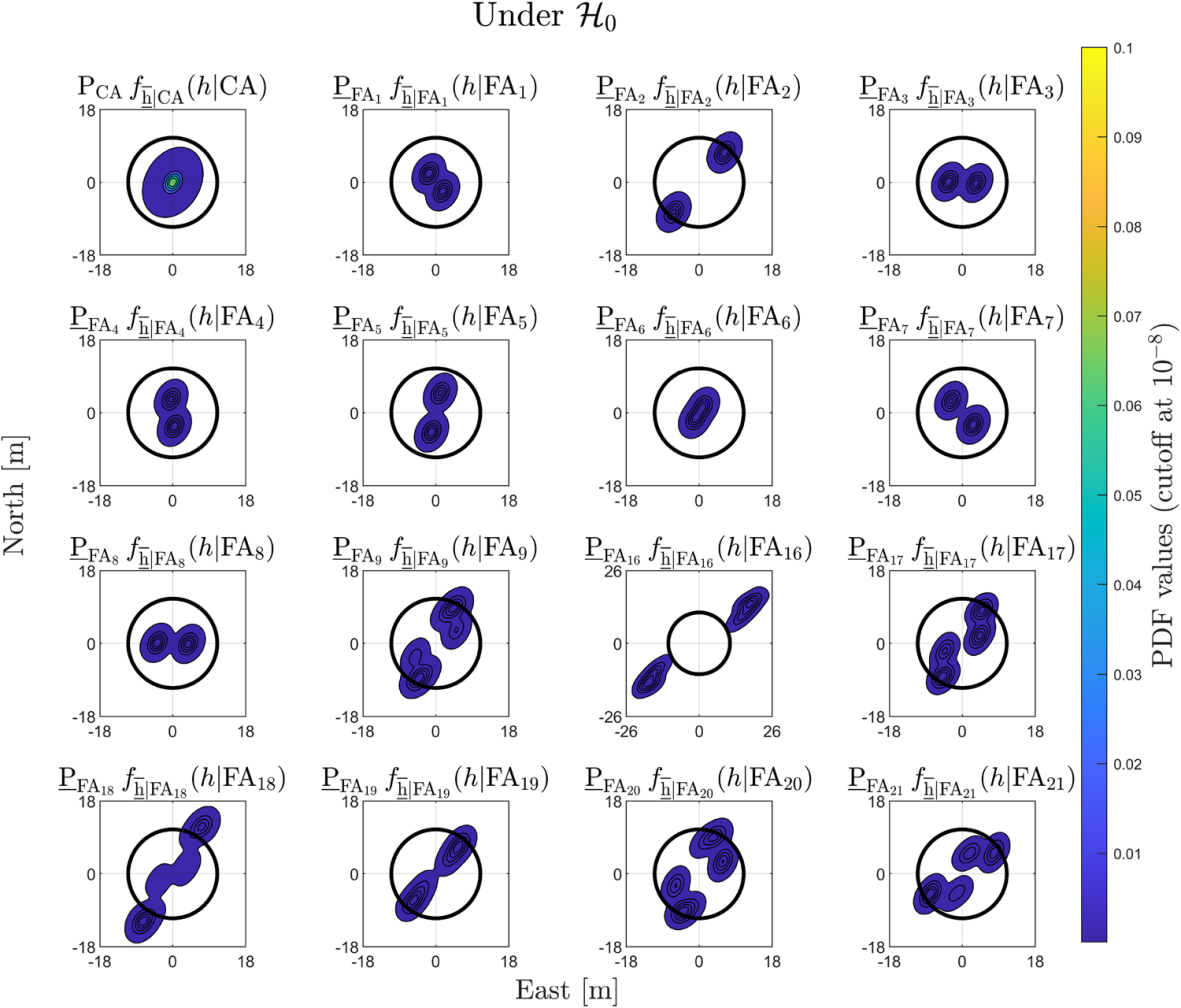
Components of $f_{\overline{\underline{\text {h}}}}\left( h|\mathcal {H}_0\right)$ for $\textsf{P}_{\text {CA}} = 1- \textsf{P}_{\text {FA}}$ and $\alpha = \textsf{P}_{\text {FA}} = 10^{-3}$, in relation with the safety region $\mathcal {B}_{\text {h}} \subset \mathbb {R}^2$ defined in (27). Examples of $\underline{\textsf{P}}_{\text {FA}_i}\,f_{\overline{\underline{\text {h}} } |\text {FA}_i}\left( h | \text {FA}_i \right)$ were selected for one-dimensional outliers affecting the pseudoranges from each individual GPS satellite ($q_i = 1$, for $i \in \{1, \dots , 8\}$), and for two-dimensional outliers involving all combinations that include GPS satellite 2 ($q_i = 2$, for $i \in \{9, 16,\dots , 21\}$). The cutoff of the probability density values was set to $10^{-8}$.

First, we analyze the components of the PDF of $\overline{\underline{\text {h}} } \in \mathbb {R}^2$ under $\mathcal {H}_0$

29 $$\begin{aligned} f_{\overline{\underline{\text {h}} }}\left( h | \mathcal {H}_0\right) = \textsf{P}_{\text {CA}}\,f_{\overline{\underline{\text {h}} }|\text {CA}}\left( h | \text {CA}\right) + \sum ^{k=36}_{i=1}\textsf{P}_{\text {FA}_i}\,f_{\overline{\underline{\text {h}} } |\text {FA}_i}\left( h | \text {FA}_i \right) , \end{aligned}$$

to highlight the factors influencing the shapes of the conditional PDFs. The significance level was set to $\alpha = \textsf{P}_{\text {FA}} = 10^{-3}$ to limit false alarms that would lead to satellite exclusion and degraded satellite geometry, while not making the Detection step (of the DIA method) too insensitive to smaller outliers. Similar values have been used in related studies (Bakker et al. 2009; Wen et al. 2021). Under the event of a CA testing decision, the PDF $f_{\overline{\underline{\text {h}} }|\text {CA}}\left( h | \text {CA}\right) = f_{\hat{\underline{\text {h}}}_0}\left( h | \mathcal {H}_0 \right)$, with $\textsf{E}\left( \hat{\underline{\text {h}}}_0|\mathcal {H}_0\right) = {\text {h}}$ where $\text {h} \in \mathbb {R}^2$ is the true horizontal position vector and $\text {Q}_{\hat{\text {h}}_0 \hat{\text {h}}_0} = \text {H}^T\text {Q}_{\hat{\text {x}}_0 \hat{\text {x}}_0} \text {H}$. For simplicity, in the simulations we set $\text {h} = 0_{2 \times 1}$. Hence, the design-matrix $\text {A}$ and vc-matrix $\text {Q}_{\text {y} \text {y}}$ are driving the shape of $f_{\overline{\underline{\text {h}} }|\text {CA}}\left( h | \text {CA}\right)$. The precision of the horizontal position components $\sigma _{\hat{\text {h}}_0,\text {east}} = 1.32~[\text {m}]$, $\sigma _{\hat{\text {h}}_0,\text {north}} = 1.53~[\text {m}]$, and the correlation coefficient is $\rho _{\hat{\text {h}}_0} = 0.25$. The contours of $f_{\overline{\underline{\text {h}} }|\text {CA}}\left( h | \text {CA}\right)$ ’weighted’ by $\textsf{P}_{\text {CA}}\,$, along with the safety region $\mathcal {B}_{\text {h}}$ are shown in Fig. 5 (first plot from the top row). Since the product $\textsf{P}_{\text {CA}}\,f_{\overline{\underline{\text {h}} }|\text {CA}}\left( h | \text {CA}\right)$ is predominantly concentrated within the safety region $\mathcal {B}_{\text {h}}$, its integral outside this region (i.e., $\mathcal {B}^c_{\text {h}}$ )–and thus its contribution to $\mathbb {P}_{\mathcal {F}_{\text {h}}}|\mathcal {H}_0$–is small.

**Fig. 6:**
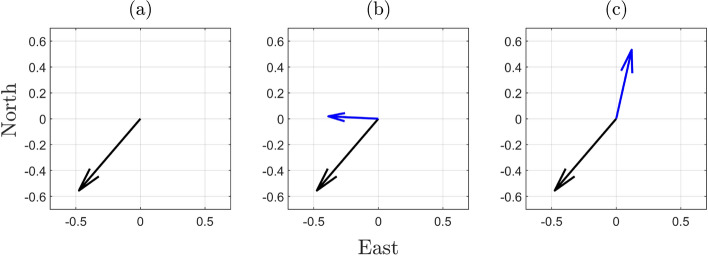
Visual representation of the columns of matrix $\text {G}_i \in \mathbb {R}^{2 \times q_i}$, for $i \in \{2,16,18 \}$, which maps $\left( \text {C}^+_{\text {t}_i}\,\underline{\text {t}}\right) \in \mathbb {R}^{q_i}$ onto the horizontal position domain; (a) Column vector $\text {G}_2 \in \mathbb {R}^{2\times (q_2=1)}$; (b) Columns of $\text {G}_{16} = [\text {G}_2~~\text {G}_{3}] \in \mathbb {R}^{2\times (q_{16}=2)}$; (c) Columns of $\text {G}_{18} = [\text {G}_2~~\text {G}_{5}] \in \mathbb {R}^{2\times (q_{18}=2)}$

Regarding the components of $f_{\overline{\underline{\text {h}} }}\left( h | \mathcal {H}_0\right)$ associated with the $\text {FA}_i$ testing decisions, we select as examples the terms $\textsf{P}_{\text {FA}_i}\,f_{\overline{\underline{\text {h}} } |\text {FA}_i}\left( h | \text {FA}_i \right)$ for: (i) one-dimensional outliers affecting, one at-a-time, the pseudorange from each individual GPS satellite with $q_i = 1$, for $i \in \{1, \dots , 8\}$, and (ii) two-dimensional outliers involving all combinations that include GPS satellite 2 (2–1, 2–3,..., 2–8.), with $q_i = 2$, for $i \in \{9, 16, \dots , 21\}$ (see Appendix 7 for the indexing of the hypotheses). These selections allow us to highlight the differences between the components $\textsf{P}_{\text {FA}_i}\,f_{\overline{\underline{\text {h}} } |\text {FA}_i}\left( h | \text {FA}_i \right)$ associated with one- and two-dimensional outlier hypotheses. Combinations of two-dimensional outliers involving GPS satellite 2 were chosen specifically because it is the only satellite located in the azimuth range $0^{\circ }-90^{\circ }$ in the skyplot shown in Fig. 3, which makes it a particularly interesting case to illustrate. We now turn our attention to the properties of the conditional PDF $f_{\overline{\underline{\text {h}} } |\text {FA}_i}\left( h | \text {FA}_i \right)$—such as orientation and multimodality—which can be studied from its expression (Teunissen 2018)

30 $$\begin{aligned} f_{\overline{\underline{\text {h}} } |\text {FA}_i}\left( h | \text {FA}_i \right) = \dfrac{\textsf{E}_{f_{\underline{\text {t}}}}\left[ f_{\hat{\underline{\text {h}}}_0}(h + \text {G}_i \text {C}^+_{\text {t}_i} \underline{\text {t}}\,|\mathcal {H}_0 )p_i(\underline{\text {t}})\right] }{\textsf{P}_{\text {FA}_i}}, \end{aligned}$$

where $\text {G}_i \in \mathbb {R}^{2 \times q_i}$ and $\text {G}_i = \text {H}^T \text {A}^+ \text {C}_i$. For every $h \in \mathbb {R}^{2}$ in the horizontal position domain, the numerator in the above equation captures two main aspects: (i) how the misclosure vector $\underline{\text {t}}\in \mathbb {R}^r$ is first transformed in the domain of $\mathbb {R}^{q_i}$ via the BLUE-inverse $\text {C}^{+}_{\text {t}_i} \in \mathbb {R}^{q_i \times r}$, and afterwards to the horizontal position domain via $\text {G}_i \in \mathbb {R}^{2 \times q_i}$; (ii) the values of the PDF $f_{\hat{\underline{\text {h}}}_0}(.)$ evaluated at $h + \text {G}_i \text {C}^+_{\text {t}_i}\underline{\text {t}}$ are averaged for all the misclosure vectors (via $\textsf{E}_{f_{\underline{\text {t}}}}\left[ .\right]$) that are in the partition $\mathcal {P}_{i \ne 0}$ (i.e., for which the indicator function $p_i(\underline{\text {t}}) = 1$). The term $\text {G}_i \in \mathbb {R}^{2 \times q_i}$ specifies which parts (or regions) of the PDF $f_{\hat{\underline{\text {h}}}_0}(.)~(=f_{\underline{\text {h}}|\text {CA}}(.))$ are evaluated through the linear combination of its columns with the coefficients given by $\left( \text {C}^{+}_{\text {t}_i} \underline{\text {t}} \right) \in \mathbb {R}^{q_i}$. Therefore, the structure of $f_{\overline{\underline{\text {h}} } |\text {FA}_i}\left( h | \text {FA}_i \right)$ is determined by the averaged shifted PDF $f_{\hat{\underline{\text {h}}}_0}\left( h + \text {G}_i \text {C}^+_{\text {t}_i} \underline{\text {t}}\,|\mathcal {H}_0\right)$ while accounting for testing decisions through the indicator function $p_i(\underline{\text {t}})$. For example, in the case of $q_i = 1$ with $i\in \{1,...,8\}$, the orientation of $f_{\overline{\underline{\text {h}} } |\text {FA}_i}\left( h | \text {FA}_i \right)$ is driven by the direction of the vector $\text {G}_i \in \mathbb {R}^{2 \times (q_i=1)}$ and how $\left( \text {C}^+_{\text {t}_i} \underline{\text {t}}\right)$ varies along its span for $\underline{\text {t}} \in \mathcal {P}_i$. Consider the conditional PDF $f_{\overline{\underline{\text {h}} } |\text {FA}_2}\left( h | \text {FA}_2 \right)$ (conditioned on the exclusion of the GPS satellite 2 observation while $\mathcal {H}_0$ is valid), which is aligned with the span of $\text {G}_2$ (see Fig. 5 and Fig. 6(a)). This shows that excluding GPS satellite 2, while $\mathcal {H}_0$ is valid, increases horizontal positioning uncertainty in its direction. The two modes of $f_{\overline{\underline{\text {h}} } |\text {FA}_2}\left( h | \text {FA}_2 \right)$ depend on how $\left( \text {C}^+_{\text {t}_2}\,\underline{\text {t}}\right)$ varies across the span of $\text {G}_2$ for $\underline{\text {t}} \in \mathcal {P}_2$. For the case where $q_i = 2$–for example, when $i \in \{16, 18\}$, which corresponds to the exclusion of GPS satellites 2–3 ($\mathcal {H}_{16}$) and 2–5 ($\mathcal {H}_{18}$)–the respective columns of $\text {G}_{16} \in \mathbb {R}^{2 \times 2}$ and $\text {G}_{18} \in \mathbb {R}^{2 \times 2}$ are shown in Figs. 6(b) and 6(c). The orientation of the conditional PDFs $f_{\overline{\underline{\text {h}} } |\text {FA}_i}\left( h | \text {FA}_i \right)$, for $i\in \{16,18\}$, is primarily determined by the direction of the columns of $\text {G}_{16}$ and $\text {G}_{18}$ associated with GPS satellite 2. Their multimodal structure is determined by the linear combinations of the columns of $\text {G}_{16}$ and $\text {G}_{18}$, where the coefficients in the vector result from $\left( \text {C}^+_{\text {t}_i} \underline{\text {t}}\right)$ for $\underline{\text {t}} \in \mathcal {P}_i$. Accounting for the computed $\underline{\textsf{P}}_{\text {FA}_i}$, Fig. 5 shows that $\underline{\textsf{P}}_{\text {FA}_{16}}\,f_{\overline{\underline{\text {h}} } |\text {FA}_{16}}\left( h | \text {FA}_{16} \right)$ and $\underline{\textsf{P}}_{\text {FA}_{18}}\,f_{\overline{\underline{\text {h}} } |\text {FA}_{18}}\left( h | \text {FA}_{18} \right)$ have the most probability density outside the safety-region $\mathcal {B}_{\text {h}}$* among all* components of $f_{\overline{\underline{\text {h}} }}\left( h | \mathcal {H}_0\right)$. For the obtained PDF $f_{\overline{\underline{\text {h}} }}\left( h | \mathcal {H}_0\right)$ we refer to Fig. 14(a) from Appendix 9.

Secondly, we compute the Level 1 components corresponding to $\mathcal {H}_0$ (see Fig. 2)

31 $$\begin{aligned} \underline{\mathbb {P}}_{\mathcal {F}_{\text {h}}}|\mathcal {H}_0 = \textsf{P}_{\text {CA}} \underline{\mathbb {P}}_{\mathcal {F}_{\text {h}}}|\text {CA} + \sum ^{k=36}_{i=1} \underline{\textsf{P}}_{\text {FA}_i} \underline{\mathbb {P}}_{\mathcal {F}_{\text {h}}}|\text {FA}_i. \end{aligned}$$

**Fig. 7:**
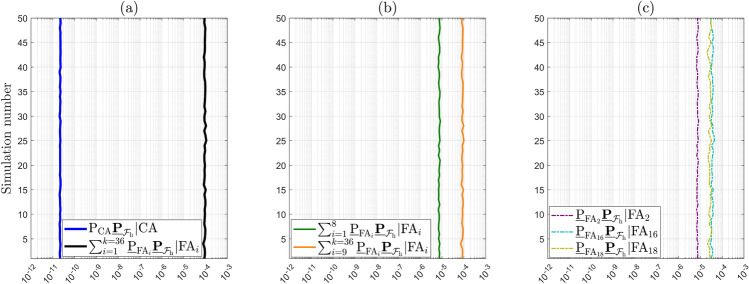
Components of $\underline{\mathbb {P}}_{\mathcal {F}_{\text {h}}}|\mathcal {H}_0$: (a) $\textsf{P}_{\text {CA}} \underline{\mathbb {P}}_{\mathcal {F}_{\text {h}}}|\text {CA}$ and $\sum ^{k=36}_{i=1} \underline{\textsf{P}}_{\text {FA}_i} \underline{\mathbb {P}}_{\mathcal {F}_{\text {h}}}|\text {FA}_i$; (b) $\sum ^{8}_{i=1} \underline{\textsf{P}}_{\text {FA}_i} \underline{\mathbb {P}}_{\mathcal {F}_{\text {h}}}|\text {FA}_i$ corresponds to outlier dimension $q_i =1$ and $\sum ^{k=36}_{i=9} \underline{\textsf{P}}_{\text {FA}_i} \underline{\mathbb {P}}_{\mathcal {F}_{\text {h}}}|\text {FA}_i$ to outlier dimension $q_i = 2$; (c) $\underline{\textsf{P}}_{\text {FA}_i} \underline{\mathbb {P}}_{\mathcal {F}_{\text {h}}}|\text {FA}_i$ for $i\in \{2,16,18\}$ correspond to the conditioning of falsely accepting hypotheses $\mathcal {H}_2$ ($q_2 = 1$ one-dimensional outlier in GPS Satellite 2), $\mathcal {H}_{16}$ ($q_{16} = 2$ two-dimensional outlier in GPS satellites 2 and 3), $\mathcal {H}_{18}$ ($q_{18} = 2$ two-dimensional outlier in GPS satellites 2 and 5)

Fig. 7 presents the results obtained from 50 independent simulation runs ($N_{\text {sim}} = 50$), which were used to empirically quantify the uncertainty in the computations (Morio and Balesdent 2016).

The uncertainty in the computation of the expected values in (18) is due to the use of simulations. Figure 7(a) shows that the component corresponding to the CA testing decision (accepting $\mathcal {H}_0$ while $\mathcal {H}_0$ is valid) has a mean value $\mu _{\text {sim}}$ of $2.1872 \cdot 10^{-11}$ across the 50 simulations. In contrast, the sum component related to *all* of the $\text {FA}_i$ testing decisions (accepting $\mathcal {H}_{i \ne 0}$ while $\mathcal {H}_0$ is valid) has a mean value $\mu _{\text {sim}}$ of $9.3056 \cdot 10^{-5}$, which not only represents the dominant contribution to $\underline{\mathbb {P}}_{\mathcal {F}_{\text {h}}}|\mathcal {H}_0$, but is also approximately *seven* orders of magnitude larger than than the component corresponding to the CA testing decision. If the summation in (31) is split into two parts–one corresponding to alternative hypotheses involving one-dimensional outliers ($q_i=1$ for $i \in \{1,..,8 \}$) and the other to those involving all the two-dimensional outliers ($q_i = 2$ for $i\in \{9,...,36\}$) –then the latter is shown in Fig. 7(b) to contribute most significantly to $\underline{\mathbb {P}}_{\mathcal {F}_{\text {h}}}|\mathcal {H}_0$. This is expected since the components $\underline{\textsf{P}}_{\text {FA}_i}\,f_{\overline{\underline{\text {h}} } |\text {FA}_i}\left( h | \text {FA}_i \right)$, for $q_i =2$, tend to exhibit ’quad’-modal behavior with a greater portion of their density lying outside the safety region–already accounted for by the ’weighting’ with $\underline{\textsf{P}}_{\text {FA}_i}$. The contribution of $\sum ^{k=36}_{i=9} \underline{\textsf{P}}_{\text {FA}_i} \underline{\mathbb {P}}_{\mathcal {F}_{\text {h}}}|\text {FA}_i$ to $\underline{\mathbb {P}}_{\mathcal {F}_{\text {h}}}|\mathcal {H}_0$ is approximately one order of magnitude larger than the one of $\sum ^{8}_{i=1} \underline{\textsf{P}}_{\text {FA}_i} \underline{\mathbb {P}}_{\mathcal {F}_{\text {h}}}|\text {FA}_i$. Taking a closer look at the two summation term $\sum ^{8}_{i=1} \underline{\textsf{P}}_{\text {FA}_i} \underline{\mathbb {P}}_{\mathcal {F}_{\text {h}}}|\text {FA}_i$, Fig. 7(c) shows that the component $\underline{\textsf{P}}_{\text {FA}_2} \underline{\mathbb {P}}_{\mathcal {F}_{\text {h}}}|\text {FA}_2$ is the largest among the group with $q_i = 1$, which is consistent with the fact that GPS satellite 2 is the only one located in the azimuth range $0^{\circ }-90^{\circ }$ (see Fig. 3). In the case of $\sum ^{k=36}_{i=9} \underline{\textsf{P}}_{\text {FA}_i} \underline{\mathbb {P}}_{\mathcal {F}_{\text {h}}}|\text {FA}_i$, with $q_i = 2$, the components $\underline{\textsf{P}}_{\text {FA}_{16}} \underline{\mathbb {P}}_{\mathcal {F}_{\text {h}}}|\text {FA}_{16}$ and $\underline{\textsf{P}}_{\text {FA}_{18}} \underline{\mathbb {P}}_{\mathcal {F}_{\text {h}}}|\text {FA}_{18}$ are most dominant - not only within that group, but also among all components with $i\in \{1,...,36\}$. Therefore, falsely excluding GPS satellite 2–or the satellite pairs 2–3 and 2–5–degrades the receiver-satellite geometry, causing the corresponding components $\underline{\textsf{P}}_{\text {FA}_i} \underline{\mathbb {P}}_{\mathcal {F}_{\text {h}}}|\text {FA}_i$ to contribute the most to $\underline{\mathbb {P}}_{\mathcal {F}_{\text {h}}}|\mathcal {H}_0$. This numerical analysis aligns with the visual interpretation discussed at Fig. 5 and the results are summarized in Table 1.

**Table 1:** Components of $\underline{\mathbb {P}}_{\mathcal {F}}|\mathcal {H}_0$. Results are obtained over $N_{\text {sim}} = 50$, from which the mean values ($\mu _{\text {sim}}$) and their standard deviation ($\sigma _{\text {sim}}$) were computed

Component	Dim. outlier ($q_i$)	*µ*_sim_	$\sigma _{\text {sim}}$ of $\mu _{\text {sim}}$
$\textsf{P}_{\text {CA}} \underline{\mathbb {P}}_{\mathcal {F}_{\text {h}}}|\text {CA}$	–	2.1872.10^−11^	0.0084.10^−11^
$\sum ^{8}_{i=1} \underline{\textsf{P}}_{\text {FA}_i} \underline{\mathbb {P}}_{\mathcal {F}_{\text {h}}}|\text {FA}_i$	1	0.7391.10^−5^	0.0057.10^−5^
$\sum ^{k=36}_{i=9} \underline{\textsf{P}}_{\text {FA}_i} \underline{\mathbb {P}}_{\mathcal {F}_{\text {h}}}|\text {FA}_i$	2	8.5665.10^−5^	0.0682.10^−5^
$\underline{\mathbb {P}}_{\mathcal {F}_{\text {h}}}|\mathcal {H}_0$	1 and 2	9.3056.10^−5^	0.0684.10^−5^

The probabilities of the testing decisions from Table 1 are shown in Table 2. As expected, the sum of the probabilities of false alarms for hypotheses with two-dimensional outliers is larger than that for hypotheses with one-dimensional outliers.

**Table 2:** Probabilities of testing decisions under $\mathcal {H}_0$. Results are obtained over $N_{\text {sim}} = 50$, from which the mean values ($\mu _{\text {sim}}$) and their standard deviation ($\sigma _{\text {sim}}$) were computed

Component	Dim. outlier ($q_i$)	*µ*_sim_	$\sigma _{\text {sim}}$ of $\mu _{\text {sim}}$
$\textsf{P}_{\text {CA}}$	–	0.9990	–
$\sum ^{8}_{i=1} \underline{\textsf{P}}_{\text {FA}_i}$	1	2.9500.10^−4^	0.4536.10^−4^
$\sum ^{k=36}_{i=9} \underline{\textsf{P}}_{\text {FA}_i}$	2	7.2140.10^−4^	0.8372.10^−4^
Total	1 and 2	1.0000	0.9522.10^−4^

#### Results under $\mathcal {H}_{i\ne 0}$

**Fig. 8:**
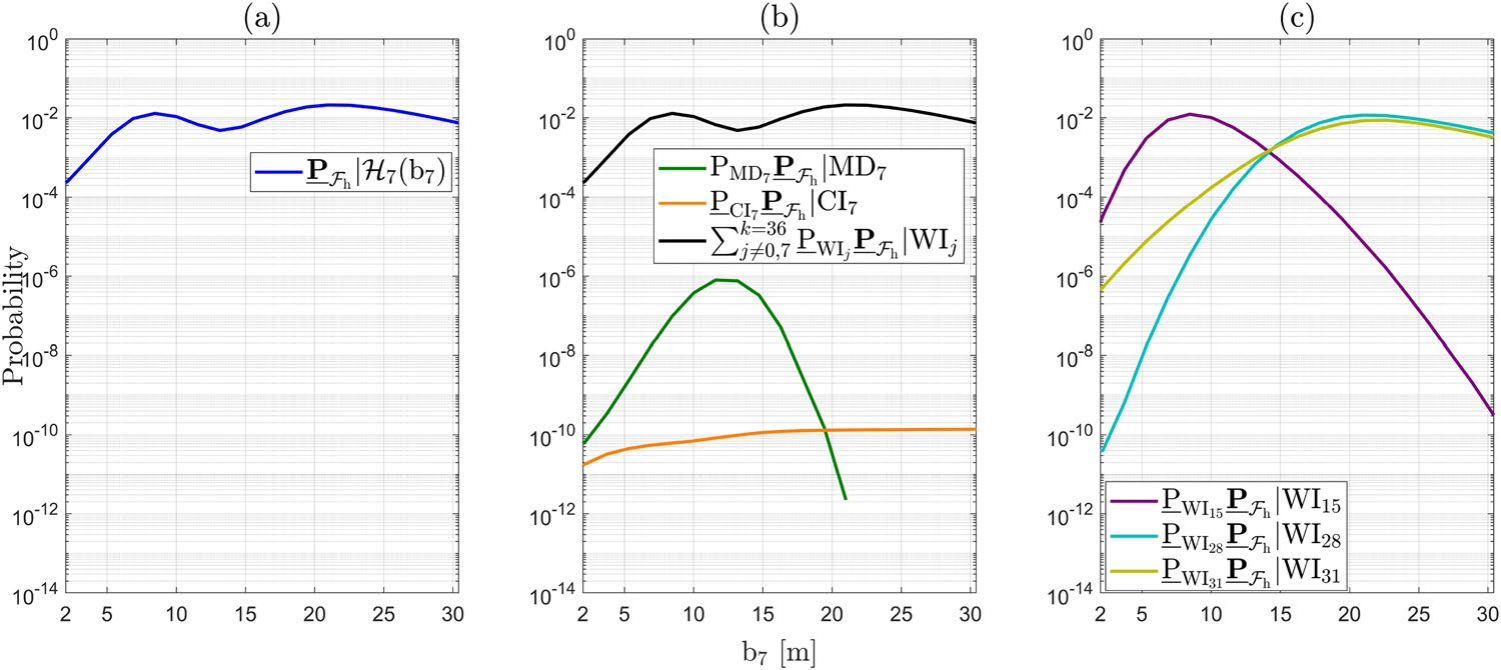
(a) Computed $\underline{\mathbb {P}}_{\mathcal {F}_{\text {h}}}|\mathcal {H}_{7}(\text {b}_{7})$ as a function of outlier size $\text {b}_7$ [m]; (b) Components of $\underline{\mathbb {P}}_{\mathcal {F}_{\text {h}}}|\mathcal {H}_{7}(\text {b}_{7})$ as a function of $\text {b}_7$ [m]: $\textsf{P}_{\text {MD}_{7}}\underline{\mathbb {P}}_{\mathcal {F}_{\text {h}}}|\text {MD}_{7}$, $\underline{\textsf{P}}_{\text {CI}_{7}}\underline{\mathbb {P}}_{\mathcal {F}_{\text {h}}}|\text {CI}_{7}$, and $\sum ^{k=36}_{j\ne 0,7} \underline{\textsf{P}}_{\text {WI}_j}\underline{\mathbb {P}}_{\mathcal {F}_{\text {h}}}|\text {WI}_j$; (c) $\underline{\textsf{P}}_{\text {WI}_j}\underline{\mathbb {P}}_{\mathcal {F}_{\text {h}}}|\text {WI}_j$ for $j\in \{15,28,31\}$ correspond to the conditioning of wrongly accepting hypotheses $\mathcal {H}_{15}$ ($q_{15} = 2$ two-dimensional outlier in GPS satellites 1 and 8), $\mathcal {H}_{28}$ ($q_{28} = 2$ two-dimensional outlier in GPS satellites 4 and 6), $\mathcal {H}_{31}$ ($q_{31} = 2$ two-dimensional outlier in GPS satellites 5 and 6)

**Fig. 9:**
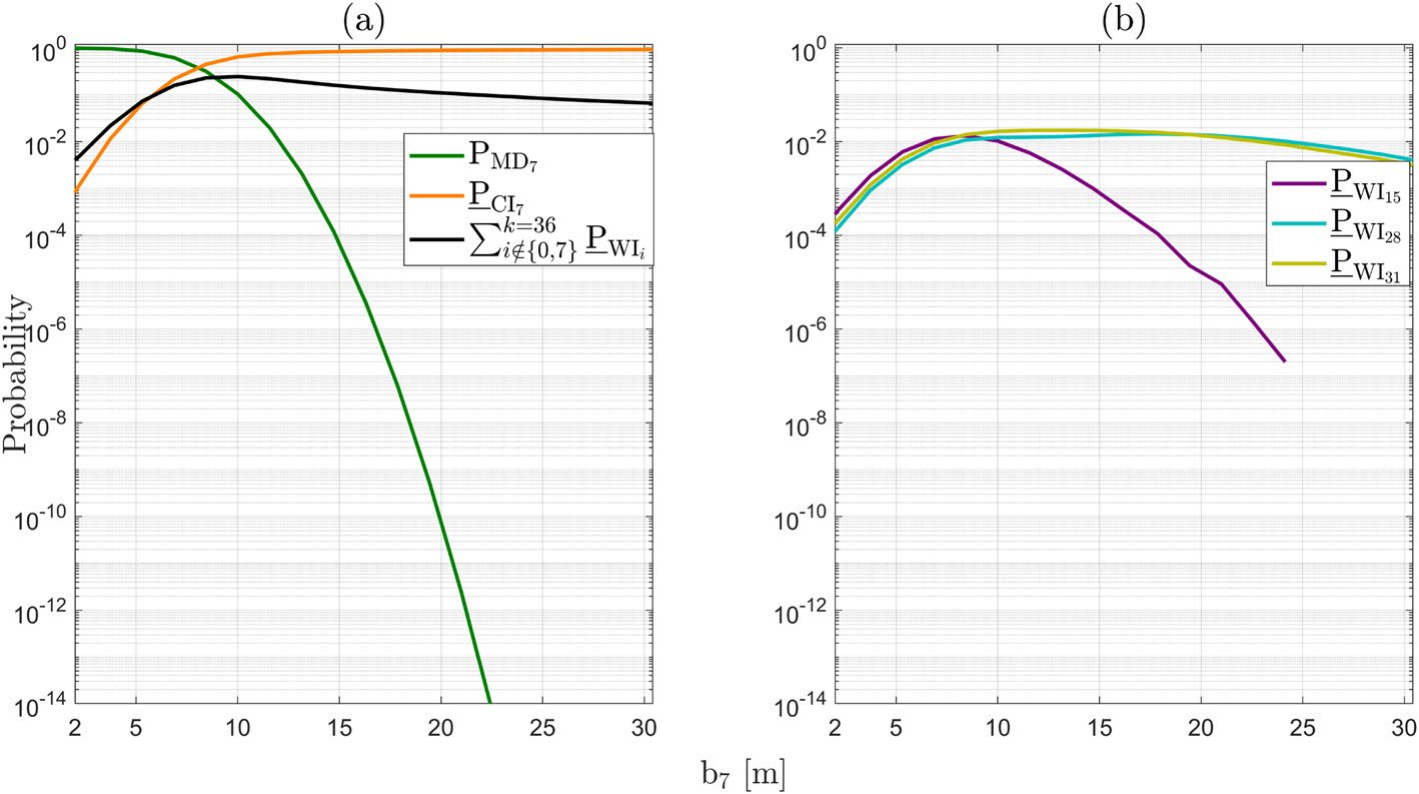
(a) Computed probabilities of testing decisions under $\mathcal {H}_7$: $\textsf{P}_{\text {MD}_{7}},~\underline{\textsf{P}}_{\text {CI}_{7}},~\sum ^{k=36}_{j \ne 0,7} \underline{\textsf{P}}_{\text {WI}_j}$ as a function of outlier size $\text {b}_7$ [m]; (b) Terms of $\sum ^{k=36}_{j \ne 0,7}\underline{\textsf{P}}_{\text {WI}_j}$ as a function of $\text {b}_7$ [m]: $\underline{\textsf{P}}_{\text {WI}_j}$ for $j\in \{15,28,31\}.$

Under the alternative hypotheses, the probability $\underline{\mathbb {P}}_{\mathcal {F}_{\text {h}}}|\mathcal {H}_{i}(\text {b}_{i})$ for $i\ne 0$ depends on the vector containing the sizes of the outliers $\text {b}_{i} \in \mathbb {R}^{q_i}$. The objective is to find its maximum (worst-case scenario). As a first example, we express the computed Level 1 components corresponding to $\mathcal {H}_{7}$ (with $q_{7} = 1$, a one-dimensional outlier in GPS Satellite 7) as follows

32 $$\begin{aligned} \underline{\mathbb {P}}_{\mathcal {F}_{\text {h}}}|\mathcal {H}_{7}(\text {b}_{7}) = \textsf{P}_{\text {MD}_{7}}\underline{\mathbb {P}}_{\mathcal {F}_{\text {h}}}|\text {MD}_{7} + \underline{\textsf{P}}_{\text {CI}_{7}}\underline{\mathbb {P}}_{\mathcal {F}_{\text {h}}}|\text {CI}_{7} + \sum ^{k=36}_{j \ne 0,7} \underline{\textsf{P}}_{\text {WI}_j}\underline{\mathbb {P}}_{\mathcal {F}_{\text {h}}}|\text {WI}_j, \end{aligned}$$

where $\textsf{P}_{\text {MD}_{7}} = \textsf{P}\left( \underline{\text {t}} \in \mathcal {P}_0\,| \mathcal {H}_7\right)$ can be computed exactly for any value of $\text {b}_{7}$. Figure 8 shows the terms in (32), averaged over $N_{\text {sim}} = 50$ independent simulation runs to obtain the empirical simulation uncertainty in the results. In Fig. 8(a), the behavior of the function $\underline{\mathbb {P}}_{\mathcal {F}_{\text {h}}}|\mathcal {H}_{7}(\text {b}_{7})$ displays a local maximum of $1.2927 \cdot 10^{-2}$ (with $\sigma _{\text {sim}} = 0.0047 \cdot 10^{-2}$) at $\text {b}_7 = 8.45~[\text {m}]$, while the global maximum is $2.1132 \cdot 10^{-2}$ (with $\sigma _{\text {sim}} = 0.0048 \cdot 10^{-2}$) for $\text {b}_7 = 21.00~[\text {m}]$. A corresponding visualization of the resulting PDF $f_{\overline{\underline{\text {h}} }}\left( h | \mathcal {H}_7\right)$ at $\text {b}_7 = 21.00~[\text {m}]$ is provided in Fig. 14(b) in Appendix 9. To gain insights into the components that contribute to the local and global maxima, the individual terms in (32) are presented in Fig. 8(b). The figure shows that $\underline{\mathbb {P}}_{\mathcal {F}_{\text {h}}}|\mathcal {H}_{7}(\text {b}_{7})$ is *completely* driven by the term $\sum ^{k=36}_{j \ne 0,7} \underline{\textsf{P}}_{\text {WI}_j}\underline{\mathbb {P}}_{\mathcal {F}_{\text {h}}}|\text {WI}_j$, which accounts for all the wrong identification testing decisions. The other two terms, $\textsf{P}_{\text {MD}_{7}}\underline{\mathbb {P}}_{\mathcal {F}_{\text {h}}}|\text {MD}_{7}$ and $\underline{\textsf{P}}_{\text {CI}_{7}} \underline{\mathbb {P}}_{\mathcal {F}_{\text {h}}}|\text {CI}_{7}$, are in the range of $10^{-11} - 10^{-6}$, while $\sum ^{k=36}_{j \ne 0,7} \underline{\textsf{P}}_{\text {WI}_j}\underline{\mathbb {P}}_{\mathcal {F}_{\text {h}}}|\text {WI}_j$ is in the range of $10^{-4} - 10^{-2}$. In the case of $\textsf{P}_{\text {MD}_{7}}\underline{\mathbb {P}}_{\mathcal {F}_{\text {h}}}|\text {MD}_{7}$, as the outlier $\text {b}_{7}$ increases, the probability density of $f_{\overline{\underline{\text {h}} }| \text {MD}_{7}}\left( h | \text {MD}_{7}\right)$ increases outside $\mathcal {B}_{\text {h}}$, while $\textsf{P}_{\text {MD}_{7}}$ decreases (i.e., the probability density of $f_{\underline{\text {t}}}(t|\mathcal {H}_7)$ decreases in the acceptance region $\mathcal {P}_0 \subset \mathbb {R}^{r=4}$). After $\textsf{P}_{\text {MD}_{7}} \underline{\mathbb {P}}_{\mathcal {F}_{\text {h}}}|\text {MD}_{7}$ reaches its maximum at $7.9663 \cdot 10^{-7}$ (with $\sigma _{\text {sim}} = 0.1025 \cdot 10^{-7}$) for $\text {b}_7 = 11.58~[\text {m}]$, its decrease is driven by $\textsf{P}_{\text {MD}_{7}}$ as its values are significantly lower than those of $\underline{\mathbb {P}}_{\mathcal {F}_{\text {h}}}|\text {MD}_{7}$. The behavior of $\textsf{P}_{\text {MD}_{7}}$ is also shown in Fig. 9(a).

In the case of the event of $\text {CI}_{7}$, the term $\underline{\textsf{P}}_{\text {CI}_{7}} \underline{\mathbb {P}}_{\mathcal {F}_{\text {h}}}|\text {CI}_{7}$ shows an increasing trend as $\text {b}_{7}$ increases, eventually approaching an almost constant behavior for $\text {b}_{7} > 20~\text {[m]}$ (around $1.3 \cdot 10^{-10}$). This occurs because the PDF $f_{\overline{\underline{\text {h}} }| \text {CI}_{7}}\left( h | \text {CI}_{7}\right)$ moves closer to the center of the safety region $\mathcal {B}_{\text {h}}$ and $\underline{\textsf{P}}_{\text {CI}_{7}} \rightarrow 1$ as $\text {b}_{7}$ increases which is also shown in Fig. 9(a).

Figure 8(c) shows the terms of $\sum ^{k=36}_{j \ne 0,7} \underline{\textsf{P}}_{\text {WI}_j}\underline{\mathbb {P}}_{\mathcal {F}_{\text {h}}}|\text {WI}_j$ that contribute most to it. These terms are $\underline{\textsf{P}}_{\text {WI}_{15}}\underline{\mathbb {P}}_{\mathcal {F}_{\text {h}}}|\text {WI}_{15}$, $\underline{\textsf{P}}_{\text {WI}_{28}}\underline{\mathbb {P}}_{\mathcal {F}_{\text {h}}}|\text {WI}_{28}$, and $\underline{\textsf{P}}_{\text {WI}_{31}}\underline{\mathbb {P}}_{\mathcal {F}_{\text {h}}}|\text {WI}_{31}$, which correspond to the wrongful identification of $\mathcal {H}_{15}$, $\mathcal {H}_{28}$, and $\mathcal {H}_{31}$, while $\mathcal {H}_7$ is valid. The corresponding probabilities of wrong identifications (related only to the testing decisions) are shown in Fig. 9(b). Each of these alternative hypotheses accounts for two-dimensional outliers ($q_{15}=q_{28}=q_{31}=2$) affecting the pseudoranges of the following pairs of GPS satellites: 1–8 ($\mathcal {H}_{15}$), 4–6 ($\mathcal {H}_{28}$), 5–6 ($\mathcal {H}_{31}$).

**Table 3:** Angles between $\mathcal {R}\left( \text {c}_{\text {t}_7} \right)$ and $\mathcal {R}\left( \text {C}_{\text {t}_j} \right)$ for $j \in \{15,28,31\}$

Subspace	$\mathcal {R}\left( \text {C}_{\text {t}_{15}} \right)$	$\mathcal {R}\left( \text {C}_{\text {t}_{28}} \right)$	$\mathcal {R}\left( \text {C}_{\text {t}_{31}} \right)$
$\mathcal {R}\left( \text {c}_{\text {t}_7} \right)$	$31.06^{\circ }$	$12.32^{\circ }$	$13.80^{\circ }$

One contributing factor to the dominant behavior of the three components in Fig. 8(c) is the angles between the subspaces spanned by $\mathcal {R}\left( \text {C}_{\text {t}_j} \right)$, for $j\in \{15, 28, 31\}$, and the subspace spanned by $\mathcal {R}\left( \text {c}_{\text {t}_7} \right)$ in the misclosure space $\mathbb {R}^{r=4}$. The smaller these angles are, the closer the corresponding subspaces are (see Table 3 and Appendix 8 for all the other angles), and hence the larger the probabilities of wrong identifications. As $f_{\underline{\text {t}}}(t | \mathcal {H}_7)$ moves along $\mathcal {R}\left( \text {c}_{\text {t}_7} \right)$ in $\mathcal {P}_7$ (as $\text {b}_7$ increases), the probability density outside $\mathcal {P}_7$ is also increasing in $\mathcal {P}_{15}$, $\mathcal {P}_{28}$, $\mathcal {P}_{31}$ up until $\text {b}_7$ reaches approximately 8.4 [m] (see the corresponding $\underline{\textsf{P}}_{\text {WI}_{j}}$ in Fig. 9(b)). After $\text {b}_7$ reaches approximately 8.4 [m], the faster decreasing behavior of the density in $\mathcal {P}_{15}$ (i.e., $\underline{\textsf{P}}_{\text {WI}_{15}}$) is due to the larger angle $\measuredangle \left( \mathcal {R}\left( \text {c}_{\text {t}_7} \right) , \mathcal {R}\left( \text {C}_{\text {t}_{15}} \right) \right)$ compared with $\measuredangle \left( \mathcal {R}\left( \text {c}_{\text {t}_7} \right) , \mathcal {R}\left( \text {C}_{\text {t}_{28}} \right) \right)$ and $\measuredangle \left( \mathcal {R}\left( \text {c}_{\text {t}_7} \right) , \mathcal {R}\left( \text {C}_{\text {t}_{31}} \right) \right)$. The second contributing factor is how the probability density of conditional PDFs $f_{\overline{\underline{\text {h}} }| \text {WI}_{j}}\left( h | \text {WI}_{j}\right)$ for $j\in \{15,28,31 \}$ varies in relation to the safety region as a function of $\text {b}_7$. For example, if the satellite pair 1–8 ($\mathcal {H}_{15}$) is wrongfully excluded while the outlier is in satellite 7, leads to $f_{\overline{\underline{\text {h}} }| \text {WI}_{7}}\left( h | \text {WI}_{7}\right)$ having a high density outside $\mathcal {B}_{\text {h}}$ in the horizontal direction corresponding to the skyplot azimuth range $270^{\circ } - 0^{\circ }$ (see Fig. 3). Hence, the dominant behavior of $\underline{\textsf{P}}_{\text {WI}_{15}}\underline{\mathbb {P}}_{\mathcal {F}_{\text {h}}}|\text {WI}_{15}$ is initially driven by $\underline{\mathbb {P}}_{\mathcal {F}_{\text {h}}}|\text {WI}_{15}$ up to its peak around $\text {b}_7 = 8.4$ [m]. Beyond that point, the behavior is governed by $\underline{\textsf{P}}_{\text {WI}_{15}}$ which decreases more rapidly than the other components in the plot as the angle $\measuredangle \left( \mathcal {R}\left( \text {c}_{\text {t}_7} \right) , \mathcal {R}\left( \text {C}_{\text {t}_{15}} \right) \right) = 31.06^{\circ }$ is larger than $\measuredangle \left( \mathcal {R}\left( \text {c}_{\text {t}_7} \right) , \mathcal {R}\left( \text {C}_{\text {t}_{28}} \right) \right) = 12.32^{\circ }$ and $\measuredangle \left( \mathcal {R}\left( \text {c}_{\text {t}_7} \right) , \mathcal {R}\left( \text {C}_{\text {t}_{31}} \right) \right) = 13.80^{\circ }$ (see Table 3). A similar reasoning can be extended to the remaining components. Therefore, wrongfully excluding these groups of satellites–while the actual outlier is present in GPS satellite 7–leads the corresponding terms $\underline{\textsf{P}}_{\text {WI}_{j}}\underline{\mathbb {P}}_{\mathcal {F}_{\text {h}}}|\text {WI}_{j}$, for $j\in \{15,28,31\}$, to dominate $\underline{\mathbb {P}}_{\mathcal {F}_{\text {h}}}|\mathcal {H}_{7}(\text {b}_{7})$ for outlier $\text {b}_7$ values around 8 and 21 [m]. An interesting point to note is that the global maximum of $\underline{\mathbb {P}}_{\mathcal {F}_{\text {h}}}|\mathcal {H}_{7}(\text {b}_{7})$ arises from the combined contribution of $\underline{\textsf{P}}_{\text {WI}_{28}}\underline{\mathbb {P}}_{\mathcal {F}_{\text {h}}}|\text {WI}_{28}$ and $\underline{\textsf{P}}_{\text {WI}_{31}}\underline{\mathbb {P}}_{\mathcal {F}_{\text {h}}}|\text {WI}_{31}$.

**Fig. 10:**
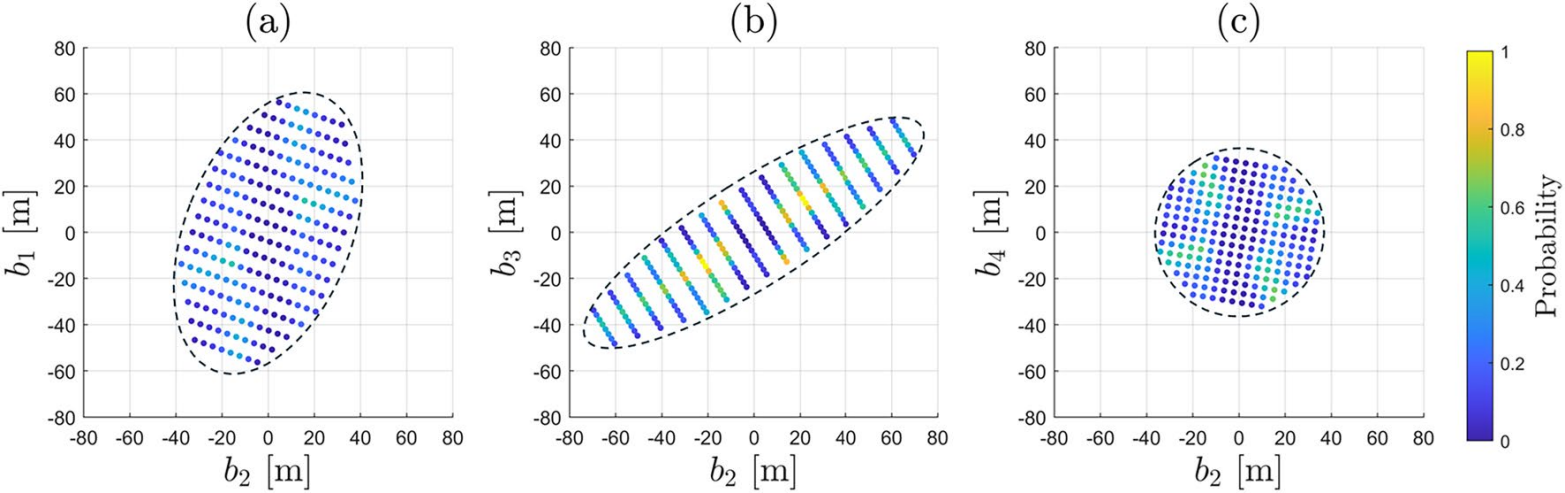
Ellipses shown with dashed lines are defined by (33), the interiors of which are then discretized; (a) $\underline{\mathbb {P}}_{\mathcal {F}_{\text {h}}}|\mathcal {H}_{9}(\text {b}_{9})$ over a grid $\text {b}_9 = [\text {b}_2~\text {b}_1]^T~\in \mathbb {R}^{q_9=2}~[\text {m}]$; (b) $\underline{\mathbb {P}}_{\mathcal {F}_{\text {h}}}|\mathcal {H}_{16}(\text {b}_{16})$ over a grid $\text {b}_{16} = [\text {b}_2~\text {b}_3]^T~\in \mathbb {R}^{q_{16}=2}~[\text {m}]$; (c) $\underline{\mathbb {P}}_{\mathcal {F}_{\text {h}}}|\mathcal {H}_{17}(\text {b}_{17})$ over a grid $\text {b}_{17} = [\text {b}_2~\text {b}_4]^T~\in \mathbb {R}^{q_{17}=2}~[\text {m}]$

As a second example, we analyze the probabilities of positioning failure under several hypotheses where $q_i = 2$, such as $\mathcal {H}_9$ (outliers in GPS Satellites 1 and 2), $\mathcal {H}_{16}$ (outliers in GPS Satellites 2 and 3), and $\mathcal {H}_{17}$ (outliers in GPS Satellites 2 and 4). The corresponding probabilities $\underline{\mathbb {P}}_{\mathcal {F}_{\text {h}}}|\mathcal {H}_{i}(\text {b}_{i})$, for $i \in \{9,16,17\}$, are displayed on two-dimensional grids in Fig. 10. These grids were obtained based on the following parametrization (Teunissen 2006)

33 $$\begin{aligned} \text {b}_i = \sqrt{\dfrac{\lambda ^2_0}{|| \Pi ^{\perp }_{\text {A}} \text {C}_i\, \text {d}||^2_{\text {Q}_{\text {y} \text {y}}}}}\,\text {d} = \sqrt{\dfrac{\lambda ^2_0}{|| \text {C}_{\text {t}_i}\, \text {d}||^2_{\text {Q}_{\text {t} \text {t}}}}}\,\text {d} \,, \end{aligned}$$

where $\lambda ^2_0$ is the non-centrality parameter of $\vert \vert \underline{\text {t}} \vert \vert ^2_{\text {Q}_{\text {t} \text {t}}} \overset{\mathcal {H}_i}{\sim } \chi ^2(r,\lambda ^2_0)$ and $\text {d} \in \mathbb {R}^{q_i = 2}$ is a unit vector which scans the unit circle in $\mathbb {R}^{q_i=2}$. As a result, $\text {b}_i$ scans an ellipse in $\mathbb {R}^{q_i=2}$ as described by $\lambda ^2_0 = || \Pi ^{\perp }_{\text {A}} \text {C}_i\, \text {b}_i||^2_{\text {Q}_{\text {y} \text {y}}}$ (see Teunissen (2006) at page 105 and Fig. 10). The non-centrality parameter $\lambda ^2_0$ is selected such that the corresponding probability of correct detection is high (i.e., close to 1), ensuring that the ellipse encompasses a representative set of vectors $\text {b}_i$. The area within these ellipses is then discretized to form 2D grids of $\text {b}_i$, over which the probabilities are computed. In all three plots in Fig. 10, the probabilities are highest (dot colors ranging from light blue to yellow) in situations where there is an approximately one-to-one relationship between the components of $\text {b}_i$’s, for $i \in \{9,16,17\}$. In contrast, the probabilities are lowest (dot colors in dark blue) when $\text {b}_2$ lies approximately in the range of –10 to 10 [m], while $\text {b}_1$, $\text {b}_3$, and $\text {b}_{4}$ vary across their entire respective ranges. Figure 10(a) illustrates the shape and orientation of the grid for $\text {b}_{9} = [\text {b}_2~\text {b}_1]^T \in \mathbb {R}^2$, corresponding to the exclusion of GPS satellites 2 and 1 from the positioning model in (21). Figure 10(b) shows that the grid of $\text {b}_{16} = [\text {b}_2~\text {b}_3]^T \in \mathbb {R}^2$ has the largest elongation in the North-East direction as GPS satellite 2 and 3 are the only ones in the azimuth range of $0^{\circ }$-$120^{\circ }$ (see Fig. 3). In the case of Fig. 10(c), the grid of $\text {b}_{17} = [\text {b}_2~\text {b}_4]^T \in \mathbb {R}^2$ is closer to a circular shape, as the elongation caused by the exclusion from the positioning model of satellites 2 and 4 is less pronounced. The maximum value for $\underline{\mathbb {P}}_{\mathcal {F}_{\text {h}}}|\mathcal {H}_{9}(\text {b}_{9})$ is attained at $4.8613 \cdot 10^{-1}$ (with $\sigma _{\text {sim}} = 0.0047 \cdot 10^{-1}$) for $\pm \text {b}_9 = [15.79~13.70]^T~[\text {m}]$. In the case of $\underline{\mathbb {P}}_{\mathcal {F}_{\text {h}}}|\mathcal {H}_{16}(\text {b}_{16})$ (Fig. 10(b)) the maximum is $9.1177 \cdot 10^{-1}$ (with $\sigma _{\text {sim}} = 0.0053 \cdot 10^{-1}$) for $\pm \text {b}_{16} = [21.04~14.68]^T~[\text {m}]$, and that of $\underline{\mathbb {P}}_{\mathcal {F}_{\text {h}}}|\mathcal {H}_{17}(\text {b}_{17})$ (Fig. 10(c)) the maximum is $5.9342 \cdot 10^{-1}$ (with $\sigma _{\text {sim}} = 0.0050 \cdot 10^{-1}$) for $\pm \text {b}_{17} = [15.35~-24.90]^T~[\text {m}]$. The maximum is determined in a similar manner for the other cases of $\underline{\mathbb {P}}_{\mathcal {F}_{\text {h}}}|\mathcal {H}_{i}(\text {b}_{i})$, for

**Fig. 11:**
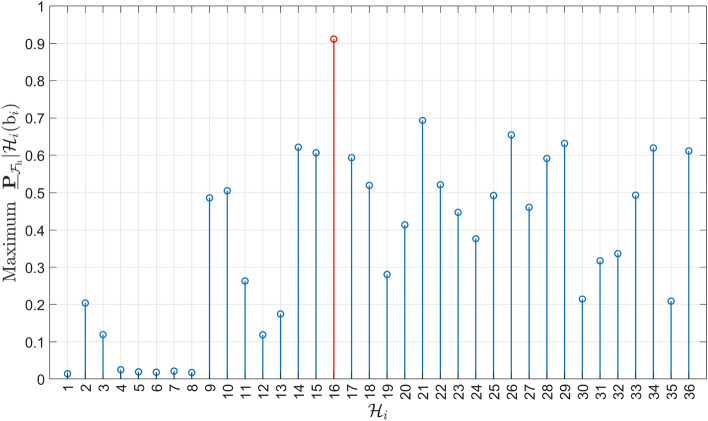
Maximum values of $\underline{\mathbb {P}}_{\mathcal {F}_{\text {h}}}|\mathcal {H}_{i}(\text {b}_{i})$ for all $i \in \{1,...,36\}$. The red stem represents $\underline{\mathbb {P}}_{\mathcal {F}_{\text {h}}}|\mathcal {H}_{16}(\text {b}_{16})$, which corresponds to two-dimensional outliers in the pseudoranges from GPS Satellites 2 and 3

$i \notin \{0,9,16,17\}$. The maximum values of $\underline{\mathbb {P}}_{\mathcal {F}_{\text {h}}}|\mathcal {H}_{i}(\text {b}_{i})$ for all $i \in \{1,...,36\}$ are shown in Fig. 11. Figure 11 shows that the maximum values of $\underline{\mathbb {P}}_{\mathcal {F}_{\text {h}}}|\mathcal {H}_{i}(\text {b}_{i})$ among the components corresponding to $q_i = 1$ (i.e., $i\in \{1,...,8\})$ are highest for $\underline{\mathbb {P}}_{\mathcal {F}_{\text {h}}}|\mathcal {H}_{2}(\text {b}_{2})$ and $\underline{\mathbb {P}}_{\mathcal {F}_{\text {h}}}|\mathcal {H}_{3}(\text {b}_{3})$. This is expected, as one-dimensional outliers at a time in GPS satellites 2 and 3–the only ones located in the azimuth range $0^{\circ }-120^{\circ }$ of the skyplot–have a greater impact on horizontal positioning performance. Since GPS satellites 2 and 3 are the only ones in the aforementioned azimuth range, outliers in their measurements have a stronger influence on the horizontal solution due to the receiver–satellite geometry. Furthermore, the maximum values of $\underline{\mathbb {P}}_{\mathcal {F}_{\text {h}}}|\mathcal {H}_{i}(\text {b}_{i})$ for $q_i = 2$ (i.e., $i\in \{9,...,36\})$ are several times larger than those for $q_i = 1$ (i.e., $i \in \{1,..,8\}$). The largest value among all alternative hypotheses corresponds to $\underline{\mathbb {P}}_{\mathcal {F}_{\text {h}}}|\mathcal {H}_{16}(\text {b}_{16})$, which is associated with the simultaneous two-dimensional outliers in the pseudoranges from GPS satellites 2 and 3 (see Fig. 3).

#### Worst-case scenario with respect to $\text {b}_i \in \mathbb {R}^{q_i}$

By using the a priori probabilities of the alternative hypotheses from Fig. 4, the computed value of $\underline{\mathbb {P}}_{\mathcal {F}_{\text {h}}}|\mathcal {H}_0$ from Table 1, and the maximum values of $\underline{\mathbb {P}}_{\mathcal {F}_{\text {h}}}|\mathcal {H}_{i}(\text {b}_{i})$ from Fig. 11, one can evaluate the upper bound in the worst-case scenario, w.r.t to $\text {b}_{i} \in \mathbb {R}^{q_i}$, from ($28$) as follows:

34 $$\begin{gathered} \underset{\breve{\mathbf{b}}}{\max}\,\breve{\underline{\mathbb{P}}}_{\mathcal{F}_{\mathrm{h}}} (\breve{\mathbf{b}}) = P({\mathcal{H}}_{0} )\underline{{\mathbb{P}}} _{{{\mathcal{F}}_{h} }} |{\mathcal{H}}_{0} \hfill \\ + \left( {\mathop {\max }\limits_{{b_{1} ,...,b_{k} }} \sum\limits_{{i = 1}}^{{k = 36}} {P({\mathcal{H}}_{i} )\underline{{\mathbb{P}}} _{{{\mathcal{F}}_{h} }} |{\mathcal{H}}_{i} (b_{i} )} } \right) + \sum\limits_{{i = 37}}^{{k^{\prime} = 255}} {P({\mathcal{H}}_{i} )} . \hfill \\ \end{gathered} $$

The numerical results are presented in Table 4 (for $\alpha = 10^{-3}$), covering a range from more conservative cases (Case 1 with $\pi = 10^{-3}$) to more optimistic ones (Case 3 with $\pi = 10^{-5}$) cf. Figure 4(b). None of the cases, when $\alpha = 10^{-3}$, approaches the level of $10^{-5}$ for the maximum $\breve{\underline{\mathbb {P}}}_{\mathcal {F}_{\text {h}}}(\breve{\textbf{b}})$, which would be needed for SAIL 4 en-route operations. If we set the level of significance to a much lower value, for example $\alpha = 10^{-5}$, the results indicate that only Case 3 (the most optimistic) would be appropriate for SAIL 4. This is because the contribution of false alarm components to $\underline{\mathbb {P}}_{\mathcal {F}_{\text {h}}} | \mathcal {H}_0$ is lower when $\alpha = 10^{-5}$, and also because the a priori probability $\textsf{P}(\mathcal {H}_0)$ is high for $\pi = 10^{-5}$, resulting in a greater contribution to (34). However, caution should be exercised when relying too heavily on the $\mathcal {H}_0$ component, particularly in safety-of-life applications.

**Table 4:** Maximum values of $\breve{\underline{\mathbb {P}}}_{\mathcal {F}_{\text {h}}}(\breve{\textbf{b}})$ and their standard deviations ($\sigma _{\text {sim}}$) for the three cases and for $\alpha = 10^{-3}$ and $\alpha = 10^{-5}$

			$\alpha = 10^{-3}$		$\alpha = 10^{-5}$	
Cases	$\pi$	$\textsf{P}(\mathcal {H}_0)$	Max. $\breve{\underline{\mathbb {P}}}_{\mathcal {F}_{\text {h}}}(\breve{\textbf{b}})$	$\sigma _{\text {sim}}$	Max. $\breve{\underline{\mathbb {P}}}_{\mathcal {F}_{\text {h}}}(\breve{\textbf{b}})$	$\sigma _{\text {sim}}$
1	10^-3^	0.99203	5.1816.10^-4^	0.0059.10^-4^	5.7009.10^-4^	0.0033.10^-4^
2	10^-4^	0.99920	1.3446.10^-4^	0.0050.10^-4^	0.5757.10^-4^	0.0019.10^-4^
3	10^-5^	0.99992	0.9700.10^-4^	0.0049.10^-4^	0.0720.10^-4^	0.0019.10^-4^

Since these results correspond to a receiver–GPS satellite geometry at a single instant of time (see Fig. 3), the next step is to investigate the results obtained over a 24-hour period, accounting for the evolution of GPS satellite positions in their orbits.

### GPS satellite geometry over 24 h period

**Fig. 12:**
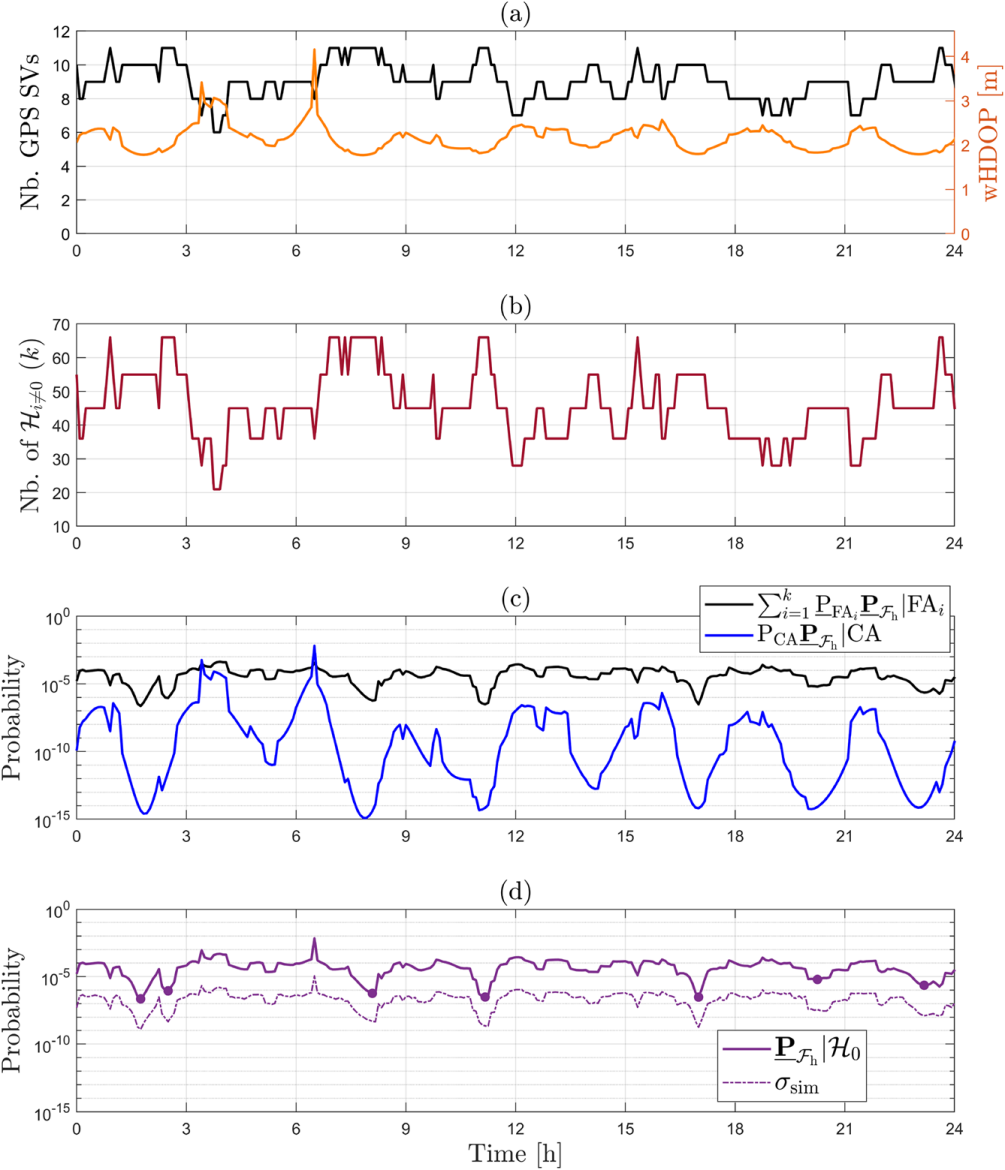
Results over time (with $\alpha = 10^{-3}$): (a) Nb. of GPS Satellites and wHDOP; (b) Nb. of alternative hypotheses $\mathcal {H}_{i\ne 0}$ (*k*); (c) Components $\textsf{P}_{\text {CA}} \underline{\mathbb {P}}_{\mathcal {F}_{\text {h}}}|\text {CA}$ and $\sum ^{k}_{i=1} \underline{\textsf{P}}_{\text {FA}_i} \underline{\mathbb {P}}_{\mathcal {F}_{\text {h}}}|\text {FA}_i$; (d) $\underline{\mathbb {P}}_{\mathcal {F}_{\text {h}}}|\mathcal {H}_{0}$, its $\sigma _{\text {sim}}$

**Fig. 13:**
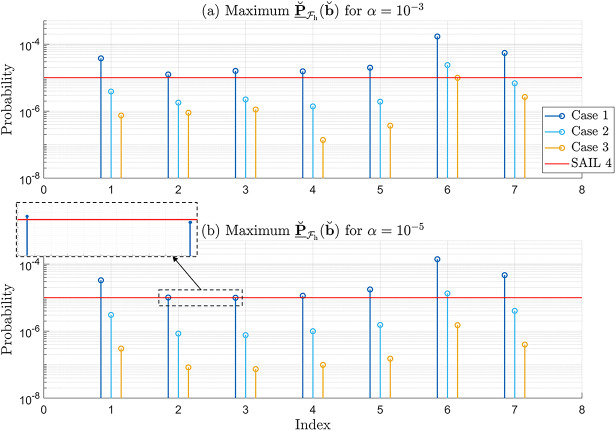
Maximum values of $\breve{\underline{\mathbb {P}}}_{\mathcal {F}_{\text {h}}} (\breve{\textbf{b}})$ for the highlighted seven points in Fig. 12. In the current plot these points are indexed from 1 to 7, left to right; (a) for $\alpha = 10^{-3}$; (b) for $\alpha = 10^{-5}$

**Fig. 14:**
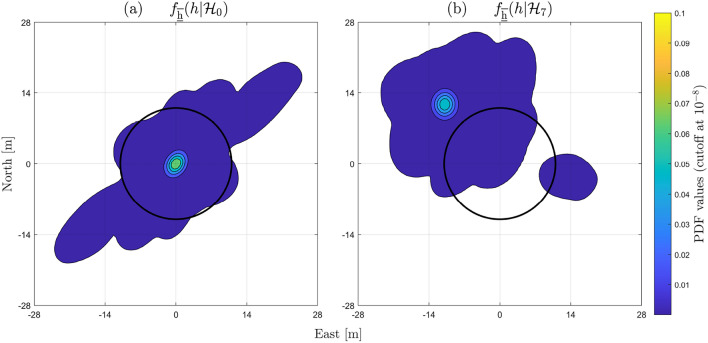
PDF of the DIA estimator $\overline{\underline{\text {h}}} \in \mathbb {R}^2$ in (26), in relation with the safety region $\mathcal {B}_{\text {h}} \subset \mathbb {R}^2$ defined in (27), and for a level of significance of $\alpha = 10^{-3}$ under the hypothesis: (a) $\mathcal {H}_0$; (b) $\mathcal {H}_7$ for $\text {b}_7 = 21~[\text {m}]$. The cutoff of the probability density values was set to $10^{-8}$

In Fig. 12(a) we show the number of observed GPS satellites (in black), using a $10^{\circ }$ elevation mask, and the weighted Horizontal Dilution of Precision (wHDOP) in orange, which is computed as $\text {wHDOP} = \sqrt{\text {trace}\left( \text {H}^T \left( \text {A}^T \text {Q}^{-1}_{\text {y} \text {y}}\text {A}\right) ^{-1}\text {H} \right) }~[\text {m}]$ based on Won (2012). The wHDOP is useful for giving an indication of the variation of the horizontal positioning precision as a function of the change in GPS satellite geometry while also taking into account the elevation-dependent weighting of the observables. Figure 12(b) shows the total number of alternative hypotheses $\mathcal {H}_{i \ne 0}$, including both one-dimensional ($q_i=1$) and two-dimensional ($q_i=2$) outliers. It can be seen that the total number of hypotheses (*k*) varies between 21 and 66. The components of $\underline{\mathbb {P}}_{\mathcal {F}_{\text {h}}}|\mathcal {H}_{0}$ are shown in Fig. 12(c), where the dominant component is $\sum ^{k}_{i=1} \underline{\textsf{P}}_{\text {FA}_i} \underline{\mathbb {P}}_{\mathcal {F}_{\text {h}}}|\text {FA}_i$ except at 3 h and 25 min ($\approx$ 3.4 h) and at 6 h and 30 min (6.5 h), when $\textsf{P}_{\text {CA}} \underline{\mathbb {P}}_{\mathcal {F}_{\text {h}}}|\text {CA}$ domaintes due to poor wHDOP. The values obtained for $\underline{\mathbb {P}}_{\mathcal {F}_{\text {h}}}|\mathcal {H}_{0}$ are shown in Fig. 12(d). When $\alpha = 10^{-3}$, even under $\mathcal {H}_0$, the values of $\underline{\mathbb {P}}_{\mathcal {F}_{\text {h}}}|\mathcal {H}_{0}$ are *above* $10^{-5}$ for approximately $78.27\%$ of the time. Redoing the computations for $\alpha = 10^{-5}$, the values of $\underline{\mathbb {P}}_{\mathcal {F}_{\text {h}}}|\mathcal {H}_{0}$ exceed $10^{-5}$ only $4.15\%$ of the time (due to the lower value of the component $\sum ^{k}_{i=1} \underline{\textsf{P}}_{\text {FA}_i} \underline{\mathbb {P}}_{\mathcal {F}_{\text {h}}}|\text {FA}_i$). We select seven points that are below $10^{-5}$ (highlighted in Fig. 12(d) by dots) and compute the remaining components of $\underset{\breve{\textbf{b}}}{\max }\,\breve{\underline{\mathbb {P}}}_{\mathcal {F}_{\text {h}}} (\breve{\textbf{b}})$, as done in (34), to verify whether the requirement is still met for the same cases from Table 4, which are restated as follows as a function of the apriori probabilities of the null hypothesis $\mathcal {H}_0$: (i) Case 1 with $\textsf{P}(\mathcal {H}_0) = 0.99203$; (ii) Case 2 with $\textsf{P}(\mathcal {H}_0) = 0.99920$; (iii) Case 3 with $\mathcal {H}_0 = 0.99992$. When $\alpha = 10^{-3}$, Fig. 13(a) shows that, for Case 1, the requirement is not met at any of the selected points. In Case 2, the requirement is not satisfied only once–at point index 6. For Case 3, the requirement is satisfied at all points, although the value at index 6 is just below the threshold. Notably, at point 6, the value of $\underline{\mathbb {P}}_{\mathcal {F}_{\text {h}}}|\mathcal {H}_{0}$ is already close to $10^{-5}$. Figure 13(b) shows the results for $\alpha = 10^{-5}$. In Case 1, only at index 3 does the maximum value fall slightly below the requirement. For Case 2, as before, the requirement is not met only once–at point index 6. For Case 3, the requirement is satisfied at all points. The results for Case 3 are more sensitive to changes in $\alpha$, which is consistent with the explanation provided for the results in Table 4.

If the requirements and guidelines are not satisfied, then appropriate changes to the measurement setup (functional and stochastic models), safety-region, or the combined parameter estimation and statistical hypothesis testing procedure, may be necessary. For instance, the new theoretical framework introduced in Teunissen (2024) shows how fit-for-purpose statistical hypothesis testing can improve the performance of DIA-estimators in terms of positioning safety.

## Computational resources

The computations in this work were performed using MATLAB R2023b with the Parallel Computing Toolbox on the Snellius Supercomputer, provided by the Dutch National e-Infrastructure. As an illustration of the computational load, evaluating the components (32) to obtain the results in Figs. 8 required computation times on the order of several minutes. These times should be seen as indicative, since they depend on the implementation details, available hardware, and programming environment.

## Summary and concluding remarks

In this contribution, we presented a general approach for performing safety analyses–such as positioning safety–based on the DIA-estimator, while accounting for multidimensional outliers in the observation model. Specifically, the study focused on misspecifications in the mean of the observation model–such as those caused by outliers in the observations. The approach includes: (i) use of the DIA-estimator and of its PDF to account for the combined uncertainty of estimation and hypothesis testing (Teunissen 2018; ii) formulation of the probability of positioning failure and of its components for an application dependent safety-region (Ciuban et al. 2025a, 2024; iii) computation of the probability of positioning failure and of its conditional components via a recently developed method (Ciuban et al. 2025b). This approach is consistent with the scenario-based safety assessment framework, which is commonly used in domains such as automated and autonomous vehicles (Riedmaier 2020; United Nations Economic Commission for Europe 2023; Gelder 2024) and UAVs (Khatiri 2023), among others.

As an illustrative case, we conducted a positioning safety analysis for a UAV, considering two scenarios involving one- and two-dimensional outlier misspecifications in the pseudorange measurements. The choice of the positioning model for the UAV, under nominal conditions, was based on the TSO certification for GPS-based UAV positioning (FAA 2017; Cozzens 2021). In the first scenario, we considered a fixed receiver-satellite geometry at a single snapshot of time (see Fig. 3) to study the shape of the DIA-estimator PDF and to gain insights into the components that contribute the most to the probability of positioning failure under $\mathcal {H}_0$. For example, the conditional components related to the False Alarm ($\text {FA}_i$) testing decisions of the DIA-estimator’s PDF (see Fig. 5) exhibited bimodal and quadrimodal structures (e.g., $f_{\overline{\underline{\text {h}}}|\text {FA}_2}(h|\text {FA}_2)$, $f_{\overline{\underline{\text {h}}}|\text {FA}_{20}}(h|\text {FA}_{20})$). The number of modes of a component $f_{\overline{\underline{\text {h}}}|\text {FA}_i}(h|\text {FA}_i) = \textsf{E}_{f_{\underline{\text {t}}}}\left[ f_{\hat{\underline{\text {h}}}_0}(h + \text {G}_i \text {C}^+_{\text {t}_i} \underline{\text {t}}\,|\mathcal {H}_0 )p_i(\underline{\text {t}})\right] /{\textsf{P}_{\text {FA}_i}}$ depends on: (i) the number of columns of the matrix $\text {G}_i \in \mathbb {R}^{2 \times q_i}$, and hence the dimension of the outlier $q_i$, and (ii) on the linear combinations of the columns of $\text {G}_i \in \mathbb {R}^{2 \times q_i}$, weighted by the coefficients $\left( \text {C}^+_{\text {t}_i}\underline{\text {t}}\right)$, for $\underline{\text {t}} \in \mathcal {P}_i$ (see Fig. 6). Notably, point (ii) depends on the hypothesis testing procedure, specifically on the partitions $\mathcal {P}_i \subset \mathbb {R}^r$ of the misclosure vector $\underline{\text {t}} \in \mathbb {R}^r$, meaning that different partitions (e.g., Teunissen 2024) can lead to different number of modes and shapes for $f_{\overline{\underline{\text {h}}}|\text {FA}_i}(h|\text {FA}_i)$.

The positioning safety analysis under $\mathcal {H}_7$ (one-dimensional outlier in GPS satellite 7) revealed that the probability $\underline{\mathbb {P}}_{\mathcal {F}_{\text {h}}}|\mathcal {H}_7(\text {b}_7)$ has a local and global maximum (see Fig. 8). These maxima were caused by the components conditioned on the Wrongful Identification (WI) testing decision $\underline{\textsf{P}}_{\text {WI}_{15}} \underline{\mathbb {P}}_{\mathcal {F}_{\text {h}}}|\text {WI}_{15}$ (pair 1–8), $\underline{\textsf{P}}_{\text {WI}_{28}} \underline{\mathbb {P}}_{\mathcal {F}_{\text {h}}}|\text {WI}_{28}$ (pair 4–6), and $\underline{\textsf{P}}_{\text {WI}_{31}} \underline{\mathbb {P}}_{\mathcal {F}_{\text {h}}}|\text {WI}_{31}$ (pair 5–6). The primary reason these components contributed the most is the strong correlation between the corresponding test statistics, combined with the degraded satellite geometry caused by the wrongful exclusion of these satellite pairs while $\mathcal {H}_7$ was valid. In general, the probability $\underline{\mathbb {P}}_{\mathcal {F}_{\text {h}}}|\mathcal {H}_i(\text {b}_i)$ can exhibit multiple local maxima as a function of $\text {b}_i \in \mathbb {R}^{q_i}$. These components can be revealed through a component-wise analysis, as carried out in this contribution and in Ciuban et al. (2024, 2025a, 2025b).

In the second scenario we have considered a 24-hours (on May 24, 2024) evolution of GPS satellites moving over an airspace region in The Netherlands for which authorization can be obtained for UAV operations (see Fig. 12). The total number of alternative hypotheses (*k*) that account for both one- and two-dimensional outliers varied between 21 and 66. Under $\mathcal {H}_0$, the computed $\underline{\mathbb {P}}_{\mathcal {F}_{\text {h}}}|\mathcal {H}_0$ was mainly driven by the component that accounts for all false alarm testing decisions $\sum ^{k}_{i=1} \underline{\textsf{P}}_{\text {FA}_i} \underline{\mathbb {P}}_{\mathcal {F}_{\text {h}}}|\text {FA}_i$. There were two cases in which $\underline{\textsf{P}}_{\text {CA}} \underline{\mathbb {P}}_{\mathcal {F}_{\text {h}}}|\text {CA} > \sum ^{k}_{i=1} \underline{\textsf{P}}_{\text {FA}_i} \underline{\mathbb {P}}_{\mathcal {F}_{\text {h}}}|\text {FA}_i$ (see Fig. 12(c)), both corresponding to the weakest satellite geometries over the 24 h, characterized by wHDOP values of 3.4 and 4.1. The results of the worst-case scenarios (w.r.t. $\text {b}_i \in \mathbb {R}^{q_i}$) for the selected points (see Fig. 12(d)) showed that Case 3 (when $\pi =10^{-5}$ in Table 4) is consistently below $10^{-5}$ (order of SAIL 4 requirement en-route operations). However, caution should be exercised when relying too heavily on the $\mathcal {H}_0$ component of the maximum $\breve{\underline{\mathbb {P}}}_{\mathcal {F}_{\text {h}}}(\breve{\textbf{b}})$, particularly in safety-of-life applications.

The *n*-dimensional DIA-estimator $\overline{\underline{\text {x}}} = \sum ^k_{i=0} \hat{\underline{\text {x}}}_i\, p_i(\underline{\text {t}})$ encompasses both statistical hypothesis testing and parameter estimation, components that are likewise fundamental to Advanced Receiver Autonomous Integrity Monitoring (ARAIM) and that are formulated under comparable modeling assumptions (Blanch et al. 2012, 2015). Different to Detection, Identification and Adaptation (DIA), ARAIM employs a testing strategy based on solution separation, primarily designed for detecting outliers in the observables (which is also the focus of the present study). Although the statistical testing procedures differ, the underlying objective is the same: assuming enough measurement redundancy formulate and use tests to select the most likely hypothesis $\mathcal {H}_i$, for $i \in \{0,...,k\}$, and provide the corresponding estimator $\hat{\underline{\text {x}}}_i \in \mathbb {R}^n$. Within the DIA-estimator framework, this corresponds to adopting a different partition structure of the misclosure space $\mathbb {R}^r$. In this sense, the DIA-estimator framework represents a class of estimators of which ARAIM’s SS-based estimator is one member. In contrast to the usual upperbounding approach of the literature, this framework also enables an explicit evaluation of the probability of positioning failure, and we show how this can be done; in principle, the same approach could be used under the ARAIM solution-separation test. Solution separation is based on the estimator difference, or ’separation’, $\hat{\underline{\text {x}}}_0 - \hat{\underline{\text {x}}}_{i \ne 0}$, which is linearly related to the misclosure vector $\underline{\text {t}} \in \mathbb {R}^r$. In ARAIM, integrity performance is typically quantified using conservative upper bounds on integrity risk (Joerger and Pervan 2016), whereas our approach evaluates the probability of positioning failure itself $\mathbb {P}_{\mathcal {F}} = \textsf{P}\left( \overline{\underline{\text {x}}} \notin \mathcal {B} \right)$ (with quantified numerical simulation uncertainty). Overall, the DIA-estimator $\overline{\underline{\text {x}}} \in \mathbb {R}^n$ provides a general framework that is also applicable to ARAIM and may offer complementary capabilities for positioning safety evaluation.

Furthermore, the type of positioning-safety analysis, as presented in this work, is broadly applicable across a range of safety-critical applications (e.g., automotive, civil aviation, rail, maritime). For example, it can support the development of positioning algorithms and systems, and their verification with respect to safety and performance requirements, as well as help in studies aimed at defining or refining such requirements. Although the positioning safety analysis was illustrated using a representative TSO-certified GPS receiver in a UAV context, the example can be extended to incorporate multisensor fusion and additional GNSS constellations (e.g., Galileo), which will be the subject of future work.

## Data Availability

No datasets were generated or analysed during the current study.

## Appendix A Indexing of the alternative hypotheses for the example in Section 4.1

The $k=36$ alternative hypotheses resulting from (25) are indexed as follows (satellites indicated between brackets below) considering the GPS satellite configuration from Fig. 3 Table 5:

- One-dimensional outliers in individual GPS satellites: $\mathcal {H}_1$ (1),..., $\mathcal {H}_8$ (8);
- Two-dimensional outliers

- Involving satellite 1: $$ \mathcal {H}_9 $$ (1–2),..., $$ \mathcal {H}_{15} $$ (1–8);
- Involving satellite 2: $$ \mathcal {H}_{16} $$ (2–3),..., $$ \mathcal {H}_{21} $$ (2–8);
- Involving satellite 3: $$ \mathcal {H}_{22} $$ (3–4),..., $$ \mathcal {H}_{26} $$ (3–8);
- Involving satellite 4: $$ \mathcal {H}_{27} $$ (4–5),..., $$ \mathcal {H}_{30} $$ (4–8);
- Involving satellite 5: $$ \mathcal {H}_{31} $$ (5–6),..., $$ \mathcal {H}_{33} $$ (5–8);
- Involving satellite 6: $$ \mathcal {H}_{34} $$ (6–7), $$ \mathcal {H}_{35} $$ (6–8);
- Involving satellite 7: $$ \mathcal {H}_{36} $$ (7–8);

## Appendix B All angles between $\angle (\mathcal {R}(c_{t_7}),\mathcal {R}(C_{t_j}))$ for all $j\ne 0,7$

**Table 5:** Angles between $\mathcal {R}\left( \text {c}_{\text {t}_7}\right)$ and $\mathcal {R}\left( \text {C}_{\text {t}_j}\right)$ for all $j \notin \{0,7\}$

Subspace	$\mathcal {R}(\text {c}_{\text {t}_{1}})$	$\mathcal {R}(\text {c}_{\text {t}_{2}})$	$\mathcal {R}(\text {c}_{\text {t}_{3}})$	$\mathcal {R}(\text {c}_{\text {t}_{4}})$	$\mathcal {R}(\text {c}_{\text {t}_{5}})$	$\mathcal {R}(\text {c}_{\text {t}_{6}})$	$\mathcal {R}(\text {c}_{\text {t}_{8}})$
$\mathcal {R}(\text {c}_{\text {t}_7})$	$74.31^{\circ }$	$72.52^{\circ }$	$80.95^{\circ }$	$68.71^{\circ }$	$87.23^{\circ }$	$27.08^{\circ }$	$69.13^{\circ }$
	$\mathcal {R}(\text {c}_{\text {t}_{9}})$	$\mathcal {R}(\text {C}_{\text {t}_{10}})$	$\mathcal {R}(\text {C}_{\text {t}_{11}})$	$\mathcal {R}(\text {C}_{\text {t}_{12}})$	$\mathcal {R}(\text {C}_{\text {t}_{13}})$	$\mathcal {R}(\text {C}_{\text {t}_{14}})$	$\mathcal {R}(\text {C}_{\text {t}_{15}})$
	$59.82^{\circ }$	$71.22^{\circ }$	$64.47^{\circ }$	$73.87^{\circ }$	$26.90^{\circ }$	$0^{\circ }$	$31.06^{\circ }$
	$\mathcal {R}(\text {C}_{\text {t}_{16}})$	$\mathcal {R}(\text {C}_{\text {t}_{17}})$	$\mathcal {R}(\text {C}_{\text {t}_{18}})$	$\mathcal {R}(\text {C}_{\text {t}_{19}})$	$\mathcal {R}(\text {C}_{\text {t}_{20}})$	$\mathcal {R}(\text {C}_{\text {t}_{21}})$	$\mathcal {R}(\text {C}_{\text {t}_{22}})$
	$68.32^{\circ }$	$61.51^{\circ }$	$68.59^{\circ }$	$23.98^{\circ }$	$0^{\circ }$	$64.77^{\circ }$	$60.88^{\circ }$
	$\mathcal {R}(\text {C}_{\text {t}_{23}})$	$\mathcal {R}(\text {C}_{\text {t}_{24}})$	$\mathcal {R}(\text {C}_{\text {t}_{25}})$	$\mathcal {R}(\text {C}_{\text {t}_{26}})$	$\mathcal {R}(\text {C}_{\text {t}_{27}})$	$\mathcal {R}(\text {C}_{\text {t}_{28}})$	$\mathcal {R}(\text {C}_{\text {t}_{29}})$
	$79.41^{\circ }$	$27.04^{\circ }$	$0^{\circ }$	$64.70^{\circ }$	$59.62^{\circ }$	$12.32^{\circ }$	$0^{\circ }$
	$\mathcal {R}(\text {C}_{\text {t}_{30}})$	$\mathcal {R}(\text {C}_{\text {t}_{31}})$	$\mathcal {R}(\text {C}_{\text {t}_{32}})$	$\mathcal {R}(\text {C}_{\text {t}_{33}})$	$\mathcal {R}(\text {C}_{\text {t}_{34}})$	$\mathcal {R}(\text {C}_{\text {t}_{35}})$	$\mathcal {R}(\text {C}_{\text {t}_{36}})$
	$63.01^{\circ }$	$13.80^{\circ }$	$0^{\circ }$	$69.05^{\circ }$	$0^{\circ }$	$25.13^{\circ }$	$0^{\circ }$

## Appendix C Plots of DIA estimator’s PDF under $\mathcal {H}_0$ and $\mathcal {H}_7$

Fig. 14(a) shows the DIA estimator’s $\overline{\underline{\text {h}}} \in \mathbb {R}^2$ PDF under $\mathcal {H}_0$, denoted $f_{\overline{\underline{\text {h}}}}(h|\mathcal {H}_0)$, as defined in (29). A distinct contribution can be observed from the conditional components $f_{\overline{\underline{\text {h}}}|\text {FA}_{16}}(h|\text {FA}_{16})$ and $f_{\overline{\underline{\text {h}}}|\text {FA}_{18}}(h|\text {FA}_{18})$, which corresponds to false alarms associated with the alternative hypothesis $\mathcal {H}_{16}$ and $\mathcal {H}_{18}$ (simultaneous outliers in the observations of GPS satellite pair 2–3 and in the pair 2–5). This is also illustrated in Fig. 5.

Figure 14(b) illustrates $f_{\overline{\underline{\text {h}}}}(h|\mathcal {H}_7)$ for an outlier size of $\text {b}_{7} = 21~[\text {m}]$, which corresponds to the global maximum of $\underline{\mathbb {P}}_{\mathcal {F}_{\text {h}}}|\mathcal {H}_{7}(\text {b}_{7})$ (see Fig. 8). At this outlier size, the dominant contributions to the shape of $f_{\overline{\underline{\text {h}}}}(h|\mathcal {H}_7)$ arise from the conditional components $f_{\overline{\underline{\text {h}}}|\text {WI}_{28}}(h|\text {WI}_{28})$ representing the wrongful identification of simultaneous outliers in GPS satellite pair 4 and 6–and $f_{\overline{\underline{\text {h}}}|\text {WI}_{31}}(h|\text {WI}_{31})$ corresponding to the wrongful identification of simultaneous outliers in satellite pair 5 and 6.

## References

[CR1] Blanch J (2015) Baseline Advanced RAIM User Algorithm and Possible Improvements. IEEE Aerosp Electr Syst 51(1):713–732

[CR2] Bakker PF, Marel HV, Teunissen PJG (2009) The Minimal Detectable Bias for Gnss Observations with a Single Receiver Setup and a Geometry-Free Model. In: Proceedings of the European Navigation Conference GNSS (ENC-GNSS), Naples, Italy

[CR3] Bakker PF, Tiberius CCJM (2017) Real-time multi-GNSS single-frequency precise point positioning. GPS Solut 21:1791–1803

[CR4] Blanch J, Walter T, Enge P, Lee Y, Pervan B, Rippl M, Spletter A (2012) Advanced RAIM User Algorithm Description: Integrity Support Message Processing, Fault Detection, Exclusion, and Protection Level Calculation. In: Proc. 25th Int. Tech. Meeting Satellite Div. Inst. Navigation (ION GNSS+), pp. 2828–2849

[CR5] Blanch J, Walter T, Enge P, Lee Y, Pervan B, Rippl M, Spletter A, Kropp V (2015) Baseline Advanced RAIM User Algorithm and Possible Improvements. IEEE Trans Aerosp Electron Syst 51(1):713–732

[CR6] Cozzens T (2021) uAvionix receives FAA order for certified drone GPS receiver. https://www.gpsworld.com/uavionix-receives-faa-order-for-certified-drone-gps-receiver/. GPS World, Aug. 5, 2021. [Online]

[CR7] Ciuban S, Teunissen PJG, Tiberius CCJM (2024) GNSS Positioning Safety: Probability of Positioning Failure and its Components. In: Proc. 37th Int. Tech. Meeting Satellite Div. Inst. Navigation (ION GNSS+), pp. 2228–2249

[CR8] Ciuban S, Teunissen PJG, Tiberius CCJM (2025a) Dependence Between Parameter Estimation and Statistical Hypothesis Testing: Positioning Safety Analysis for Automated/Autonomous Vehicles. IEEE Trans . Intell . Transp . Syst . 26(4):5509–5521

[CR9] Ciuban S, Teunissen PJG, Tiberius CCJM (2025b) A Method to Compute the Probability of Positioning Failure for Vehicles in the Context of Dependence Between Parameter Estimation and Statistical Hypothesis Testing. IEEE Trans. Vehicular Technol. Accepted for Publication (30th May 2025) 74(10):15238-15253

[CR10] DGCC: The Delft Approach for the Design and Computation of Geodetic Networks. In: Forty Years of Thought: Anniversary Edition on the Occasion of the 65th Birthday of Professor W. Baarda vol. 1, pp. 202–274 (1984)

[CR11] EASA: National Aviation Authority drone website reference. https://www.easa.europa.eu/en/domains/civil-drones/naa. [Online]

[CR12] Euler HJ, Goad CC (1991) On optimal filtering of GPS dual frequency observations without using orbit information. Bull Geod 65(2):130–143

[CR13] EUSPA: Report on aviation and drones: User needs and requirements. Technical report (2023)

[CR14] FAA: Technical Standard Order C-145e: Airborne Navigation Sensors Using the Global Positioning System Augmented by the Satellite Based Augmentation System (SBAS). Technical report (2017)

[CR15] Gelder E et al (2024) TNO Street Wise: Scenario-Based Safety Assessment of Automated Driving Systems. Technical report, Netherlands Organisation for Applied Scientific Research (TNO)

[CR16] Imparato D (2016) GNSS-based Receiver Autonomous Integrity Monitoring for Aircraft Navigation. Ph.D. Thesis, Delft University of Technology

[CR17] Joerger M, Pervan B (2016) Fault Detection and Exclusion Using Solution Separation and Chi-squared ARAIM. IEEE Trans Aerosp Electron Syst 52(2):726–742

[CR18] Johnston G, Riddell A, Hausler G (2017) The International GNSS Service. In: Teunissen PJG, Montenbruck O (eds) Springer Handbook of Global Navigation Satellite Systems. Springer, Cham, pp 967–982

[CR19] Khatiri S (2023)Simulation-based Test Case Generation for Unmanned Aerial Vehicles in the Neighborhood of Real Flights. In: Proc. IEEE Conf. Softw. Test. Verif. Valid

[CR20] Kahn H, Marshall AW (1953) Methods of Reducing Sample Size in Monte Carlo Computations. J Oper Res Soc Am 1(5):263–278

[CR21] Koch K-R (2017) Expectation Maximization algorithm and its minimal detectable outliers. Stud Geophys Geod 61(1):1–18

[CR22] Liu B, Gao Y, Gao Y, Wang S (2022) HPL calculation improvement for Chi-squared residual-based ARAIM. GPS Solutions **26**(45)

[CR23] Lehmann R, Lösler M (2016) Multiple Outlier Detection: Hypothesis Tests versus Model Selection by Information Criteria. J. Surv. Eng. **142**(4)

[CR24] Liu S, Wang K, Abel, D (2023) Robust state and protection-level estimation within tightly coupled GNSS/INS navigation system. GPS Solutions **27**(111)

[CR25] Morio J, Balesdent M (2016) Estimation of Rare Event Probabilities in Complex Aerospace and Other Systems - A Practical Approach. Elsevier B.V, Amsterdam

[CR26] Maaref M, Khalife J, Kassas ZM (2021) Aerial Vehicle Protection Level Reduction by Fusing GNSS and Terrestrial Signals of Opportunity. IEEE Trans Intell Transp Syst 22(9):5976–5993

[CR27] Odijk D (2017) Positioning Model. In: Teunissen, P.J.G., Montenbruck, O. (eds.) Springer Handbook of Global Navigation Satellite Systems, p. 617. Springer, Cham . Chap. 21

[CR28] Riedmaier S (2020) Survey on Scenario-Based Safety Assessment of Automated Vehicles. IEEE Access 8:87456–87477

[CR29] Rubinstein RY, Kroese DP (2008) Simulation and the Monte Carlo Method, 2nd edn. Wiley Series in Probability and Statistics. Wiley, Hoboken, NJ

[CR30] Rubinstein RY (1999) The cross-entropy method for combinatorial and continuous optimization. Methodol Comput Appl Prob 1:127–190

[CR31] Special Committee 159, R.T.C.A.: Minimum Operational Performance Standards (MOPS) for Global Positioning System/Satellite-Based Augmentation System Airborne Equipment. DO-229F, p. 15. Radio Technical Commission for Aeronautics, Washington, D.C. (2020)

[CR32] Teunissen PJG (1990) Nonlinear least squares Manuscr Geod 15(3):137–150

[CR33] Teunissen PJG (1990) Quality control in integrated navigation systems. IEEE Aerosp Electron Syst Mag 5(7):35–41

[CR34] Teunissen PJG (2006) Testing Theory: An Introduction, 2nd edn. Mathematical Geodesy and Positioning. Delft University Press, Delft

[CR35] Teunissen PJG (2017) Batch and Recursive Model Validation. In: Teunissen, P.J.G., Montenbruck, O. (eds.) Springer Handbook of Global Navigation Satellite Systems, pp. 687–720. Springer, Cham . Chap. 24

[CR36] Teunissen PJG (2018) Distributional theory for the DIA method. J Geod 92(1):59–80

[CR37] Teunissen PJG (2024) On the optimality of DIA-estimators: theory and applications. J Geod 98(43):1–26

[CR38] uAvionix: truFYX TSO GPS Receiver for Unmanned Aerial Systems: Product Details and Specifications. https://uavionix.store/unmanned-navigation/trufyx-tso. [Online]

[CR39] United Nations Economic Commission for Europe: New Assessment/Test Method for Automated Driving (NATM) Guidelines for Validating Automated Driving Systems (ADS). Report (2023)

[CR40] Won DH (2012) Weighted DOP With Consideration on Elevation-Dependent Range Errors of GNSS Satellites. IEEE Trans Instrum Meas 61(12):3241–3250

[CR41] Wen W, Meng Q, Hsu L-T (2021) Integrity Monitoring for GNSS Positioning via Factor Graph Optimization in Urban Canyons. In: Proceedings of the 34th International Technical Meeting of the Satellite Division of The Institute of Navigation (ION GNSS+), pp. 1508–1515

[CR42] Working Group C: ARAIM Technical Subgroup Milestone 3. Technical report, EU-US Cooperation on Satellite Navigation, Working Group C (2016)

[CR43] Yu Y, Yang L, Shen Y (2024) An extended w-test for outlier diagnostics in linear models. J Geod 98(58):1–21

[CR44] Yu Y, Yang L, Shen Y, Sun N (2023) A DIA method based on maximum a posteriori estimate for multiple outliers. GPS Solut 27(199):1–14

[CR45] Zaminpardaz S, Teunissen PJG (2023) Detection-only versus detection and identification of model misspecifications. J Geod 97(55):1–19

[CR46] Zaminpardaz S, Teunissen PJG, Tiberius CCJM (2019) Risking to underestimate the integrity risk. GPS Solut 23(29):1–16

[CR47] Zaminpardaz S, Teunissen PJG, Tiberius CCJM (2020) A risk evaluation method for deformation monitoring systems. J Geod 94(28):1–15

